# Statins for major depressive disorder: A systematic review and meta-analysis of randomized controlled trials

**DOI:** 10.1371/journal.pone.0249409

**Published:** 2021-03-30

**Authors:** Riccardo De Giorgi, Franco De Crescenzo, Nicola Rizzo Pesci, Marieke Martens, Wendy Howard, Philip J. Cowen, Catherine J. Harmer

**Affiliations:** 1 Department of Psychiatry, Warneford Hospital, University of Oxford, Oxford, Oxfordshire, United Kingdom; 2 Oxford Health NHS Foundation Trust, Warneford Hospital, Oxford, Oxfordshire, United Kingdom; University of British Columbia, CANADA

## Abstract

**Background:**

The burden of depressive disorder is large and new treatment approaches are required. Repurposing widely available drugs such as statins may be a time- and cost-effective solution. Statins have anti-inflammatory and anti-oxidant properties which have been shown to be relevant to the pathophysiology of depression. This study assesses the efficacy, acceptability, tolerability, and safety of statins in major depressive disorder.

**Methods:**

Our study is an update and extension of a previous meta-analysis published in 2016 by Salagre et al. We performed a systematic review (PubMed/MEDLINE, Cochrane CENTRAL, ISI Web of Science, CINAHL, and ClinicalTrials.gov until the 1^st^ September 2020) and meta-analysis of randomized controlled trials using any statin against placebo or any other statin in the treatment of major depressive disorder. Our primary efficacy outcome measure was the mean value on any standardized scale for depressive symptoms at 8 weeks of treatment. We also calculated outcomes for efficacy, response, and remission at 2, 4, and 12 weeks, as well as acceptability (dropouts for any cause), tolerability (dropouts due to any adverse event), and safety (any adverse event) outcomes at the studies’ endpoints. Furthermore, we conducted an exploratory network meta-analysis for the primary efficacy outcome to identify potential differences between statins.

**Results:**

We retrieved five randomized controlled trials meeting our inclusion criteria: four used a statin in addition to an antidepressant and compared it to placebo plus antidepressant, and one compared two statins alone. and one comparing one statin with another. Statins compared to placebo in addition to antidepressants were efficacious at 8 weeks (N = 255, SMD = -0.48, 95% CI = -0.74 to -0. 22) and 12 weeks (N = 134, SMD = -0.47, 95% CI = -0.89 to -0.05, moderate certainty) with no difference for acceptability, tolerability, and safety (low certainty). An exploratory network meta-analysis suggested that the most lipophilic statins, especially simvastatin, could be more efficacious than less lipophilic or hydrophilic molecules.

**Conclusions:**

This systematic review suggests the efficacy, acceptability, tolerability, and safety of statins in addition to antidepressants in patients with major depressive disorder. Further clinical trials in different settings are required to test this result.

**Trial rgistration:**

PROSPERO registration: CRD42020170938.

## Introduction

### The burden of depression

Major depressive disorder is a leading cause of disability worldwide [1] characterized by low mood, anhedonia, feelings of worthlessness and hopelessness, and disturbances of sleep, appetite, and libido. Traditional antidepressants work in the main by modulating monoamine levels in the synaptic cleft [2] and are burdened by high rates of non-response. In the Sequenced Treatment Alternatives to Relieve Depression (STAR*D) study, just under half of the patients responded to first-line pharmacological therapy and one-third still experienced significant symptoms after four treatment steps over one year of treatment [3]. In randomized placebo-controlled trials of antidepressants for the acute treatment of major depression, the placebo response rate ranges between 35% and 40% [4] versus 50% to 60% for antidepressants [5]. Therefore, the need for new antidepressant drugs is compelling, but drug development in this therapeutic area is challenging and several pharmaceutical companies have disinvested from it [6]. Repurposing currently available medications to target alternative pathways implicated in depression may provide a solution to this problem [7, 8].

### Statins in depression

#### Biological mechanisms of antidepressant response

The 3-hydroxy-3-methylglutaryl-Coenzyme A reductase inhibitors or statins are a class of anti-cholesterolemic agents largely used for the prevention and treatment of cardiovascular and metabolic disorders and their complications [9]. These medications have significant anti-inflammatory and antioxidant effects that are rapid [10] and independent of their lipid-lowering properties [11]. In fact, numerous biological mechanisms support the antidepressant potential of statins. *In vitro* studies have shown that statin-mediated cholesterol depletion alters 5 hydroxytryptamine (5HT)_1a_-receptor dynamics [12], while animal studies have reported that statins augment the serotonergic effects of some antidepressants [13] and increase hippocampal 5HT [14] and brain-derived neurotrophic factor [15–17] levels. Moreover, statins reduce depressive-like behaviors in rats by counteracting microglial and astrocyte activation as well as cytokine release in the central nervous system via inhibition of the nuclear factor-kB pathway and subsequent interleukin (IL)-1B, IL-6, and tumor necrosis factor-α secretion [18–20]; similarly, they offset the peripheral effects of IL-6 and IL-18 in humans [21]. The depressogenic effect of oxidative stress in the brain appears reduced both directly [22] and via peroxisome proliferator-activated receptor-γ activity and decreased nitrous oxide levels [23] by statins. Statins also normalize high fat diet-mediated changes in the endocannabinoid system whilst improving depressive-like behaviors in aspartate receptor antagonism [24] and the PI3k/AKT/GSK-3b/mTor signalling pathway [25], which may also mediate the antidepressant of action of ketamine.

#### Human studies and aim of the review

**Fig 1:**
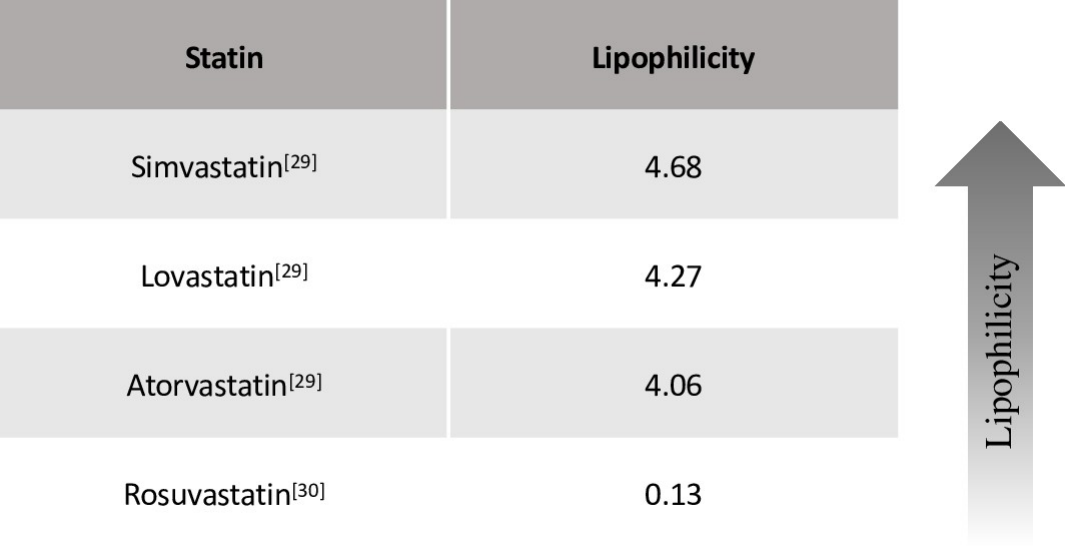
Main statins’ lipophilicity. Lipophilicity is measured as C log P octanol/water, i.e. higher values correspond to higher lipophilicity. Note that simvastatin, lovastatin, and atorvastatin [29] are considered lipophilic statins, whereas rosuvastatin [30] is a highly hydrophilic statin.

In view of their pleiotropy, well-established safety profile [26], and differential capacity to penetrate the brain parenchyma according to their lipophilicity [27] (Fig 1), the potential therapeutic use of statins has been extensively studied in depression [28].

Despite earlier conflicting reports about the impact of statins on mood [31, 32], later meta-analyses of observational [33] and interventional [34] studies, including some investigating many other anti-inflammatories [35, 36], have indeed suggested that statins may have clinically meaningful antidepressant effects. These previous studies, however, often included heterogenous populations of depressed and non-depressed participants and only pooled results for few outcome measures; moreover, other trials may have been published since.

Thus, the aim of our systematic review and meta-analysis was to investigate the recent literature to assess the effect of statins on several outcomes in patients with major depressive disorder.

## Materials and methods

We conducted a comprehensive literature search of the PubMed/MEDLINE, Cochrane CENTRAL, ISI Web of Science, CINAHL, and ClinicalTrials.gov databases from the date of inception until the 1^st^ September 2020, including non-English language articles. References of the included papers were manually screened for further relevant material. We contacted the corresponding authors to obtain information about unpublished or incomplete trials. The search algorithm (see S1 Text in S1 File) combined all the relevant terms for statins, depression, and antidepressants.

The protocol for this review was registered on PROSPERO international prospective register of systematic reviews with reference CRD42020170938 and is available at https://www.crd.york.ac.uk/PROSPERO/display_record.php?RecordID=170938.

### Types of study included

All randomized controlled trials comparing any statin with placebo or any other statin in the treatment of major depressive disorder were included. Head-to-head comparisons between statins were considered in order to investigate possible differences between statins’ effect, in view of their variable lipophilicity and thus brain penetration. Quasi-randomized trials, such as those allocating by using alternate days of the week, were excluded. For trials with a cross-over design, only results from the first randomisation period were considered.

### Population

Patients aged 12 years or older, of both sexes, with a primary diagnosis of major depression according to the diagnostic and statistical manual of mental disorders (DSM)-III to DSM-5, international classification of diseases (ICD)-10, Feighner criteria, or Research Diagnostic criteria were included. Studies using ICD-9 were excluded as this system does not use operationalized criteria. A concurrent secondary diagnosis of another psychiatric or medical disorder was not considered as exclusion criteria; however, a concurrent primary diagnosis of another psychiatric disorders was an exclusion criterion.

### Intervention, comparator

Any clinical trial using any statin either alone or in addition to an antidepressant in major depressive disorder in comparison to placebo or any other statin was included.

### Outcome(s)

For the efficacy, response, and remission outcomes we calculated results at the following endpoints (or their closest timepoints in a contiguous range): 2 weeks (1–2 weeks), 4 weeks (3–5 weeks), 8 weeks (6–10 weeks), 12 weeks (11–14 weeks). For the acceptability, tolerability, and safety outcomes we calculated results at the studies’ endpoints.

Our primary efficacy outcome measure was the mean value on the Hamilton Depression Rating Scale (HDRS), Montgomery-Åsberg Depression Rating Scale (MADRS), Beck Depression Inventory (BDI), or any other standardized scale for depressive symptoms at 8 weeks of treatment.

1. Our secondary outcomes included:
  - efficacy measured as mean value on any standardized scale for depressive symptoms at 2 weeks, 4 weeks, and 12 weeks of treatment;
  - efficacy as response measured as 50% reduction on any standardized scale for depressive symptoms at 2 weeks, 4 weeks, 8 weeks, and 12 weeks of treatment;
  - efficacy as remission measured as depression score below a pre-specified threshold on any standardized scale for depressive symptoms (e.g. <7 for HDRS) at 2 weeks, 4 weeks, 8 weeks, and 12 weeks of treatment;
  - acceptability measured as number of participants discontinuing treatment (dropouts) due to any cause at the studies’ endpoints;
  - tolerability measured as number of participants discontinuing treatment (dropouts) due to adverse events at the studies’ endpoints;
  - safety measured as number and type of adverse events at the studies’ endpoints.

### Data extraction

Two researchers (RDG, NRP) independently screened titles and abstracts for relevance and assessed the full texts for eligibility. Disagreements were discussed with a third researcher (FDC) and resolved by consensus. We used a structured data extraction form to ensure consistency of appraisal for each study. For the included studies, we extracted data about authors’ names, year of publication, study design, sample size and characteristics, intervention and comparison details, length of follow-up, primary and secondary outcome measures of interest with point estimates.

We assessed the risk of bias (RoB) of the included studies using the “RoB 1: a revised tool for assessing risk of bias in randomized trials” described in the Cochrane Collaboration Handbook(38) as a reference guide. The quality assessment was performed by two independent raters (RDG, FDC) and disagreements were discussed with another member of the review group (NRP) and resolved by consensus.

### Statistical analysis

Data was recorded in an Excel spreadsheet and analyzed using STATA v16 software [37]. Data from depression rating scales were analyzed as continuous data using standardized mean difference (SMD, as different rating scales were used) with 95% confidence intervals (CI), employing a random-effects model, which is more conservative than fixed-effects models. In interpreting SMD values, we considered SMD ‘small’ if <0.40, ‘moderate’ from 0.40 to 0.70, and ‘large’ if >0.70(38). All the other quantitative data (e.g. number of participants discontinuing treatment) were analyzed as dichotomous data using relative risk (RR) with 95% CI using random-effects meta-analysis. Non-quantitative data (e.g. type of adverse events) were presented descriptively. Heterogeneity between studies was investigated through the I^2^, t^2^, and *p-value* statistic and by visual inspection of the forest plots. If at least 10 studies were available, we would use the funnel plot and Egger’s test to detect publication bias [38].

In order to test whether statins with higher lipophilicity have a differential effect on depressive symptoms, we performed an exploratory network meta-analysis. This incorporated indirect comparisons with direct comparisons using random-effects network meta-analysis within a frequentist framework using STATA v16 software [37]. We reported the results of network meta-analyses for the primary efficacy outcome at study endpoint in a league table with effect sizes (SMD) and their 95% CIs.

## Results

### Study selection

**Fig 2:**
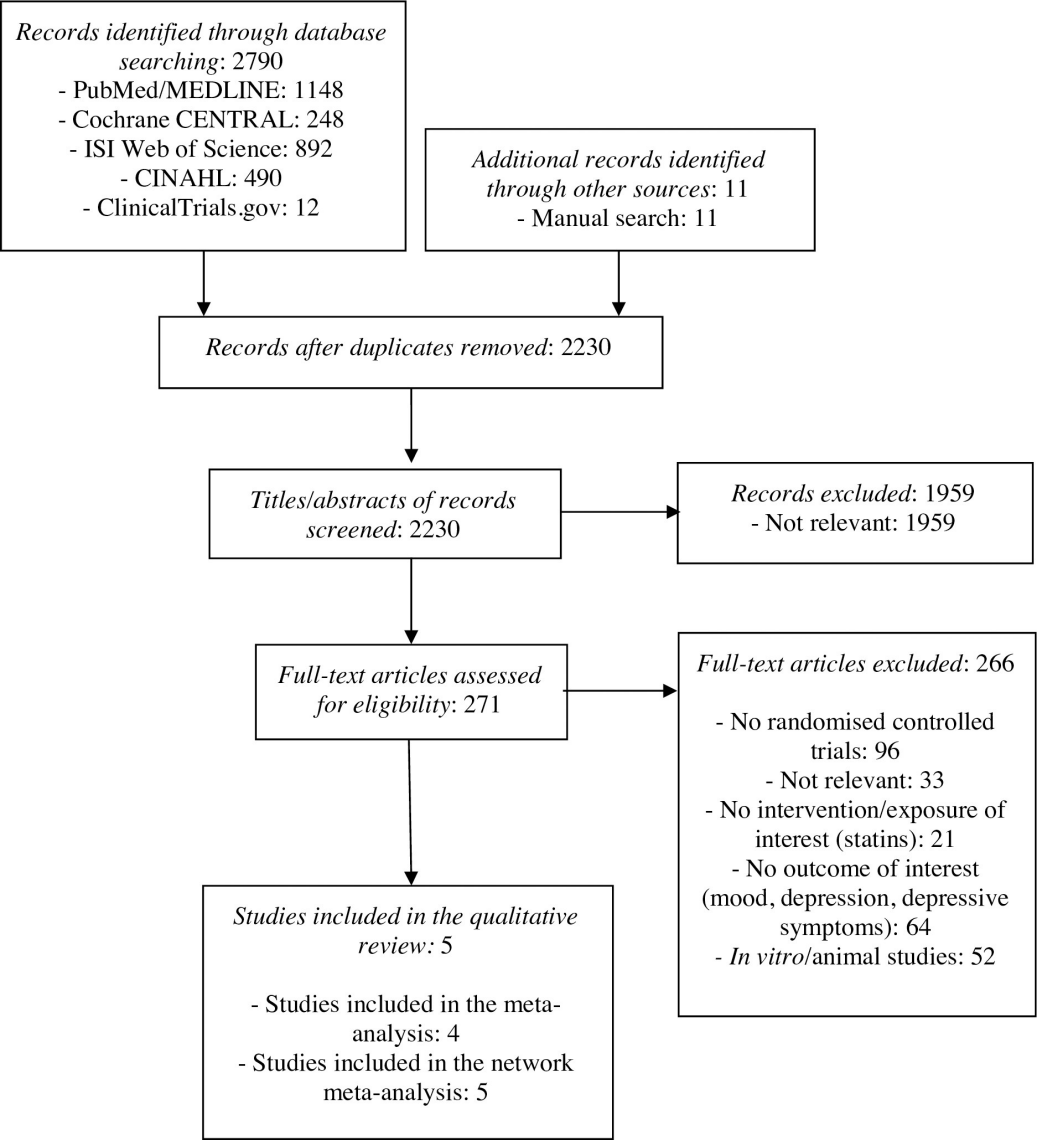
PRISMA flow chart.

The literature search was reported according to the Preferred Reporting Items for Systematic Reviews and Meta-Analyses (PRISMA) guideline (Fig 2) and retrieved 2790 records from electronic databases (PubMed/MEDLINE: 1148, Cochrane CENTRAL: 248, ISI Web of Science: 892, CINAHL: 490, ClinicalTrials.gov: 12) and further 11 papers from the manual search.

After the duplicates were removed, 2230 titles and abstracts were screened according to the criteria described in the Method section, of which 1959 were excluded due to lack of relevance. The remaining 271 articles were assessed in full: 5 were randomized controlled trials and therefore included in the qualitative synthesis. Of these, 4 could be included in the meta-analysis, and 5 were part of an exploratory network meta-analysis for efficacy.

### Study characteristics

The characteristics of the 5 retrieved trials [39–43] were reported in Table 1.

**Table 1:** Study characteristics.

Study ID	Study design	Population	Intervention	Comparator	Follow-up	Primary outcome measure
Abbasi 2015 [43]	RCT	58 post-CABG MDD patients, 18-50yo, baseline mild to moderate depression (HDRS-17 ≤ 19)	Simvastatin 20mg	Atorvastatin 20mg	6 weeks	HDRS-17
Berk 2020 [42]	RCT, placebo-controlled	90 MDD patients, 15-25yo, baseline moderate to severe depression (MADRS ≥ 20)	TAU + rosuvastatin 10mg	TAU + placebo	12 weeks	MADRS
Ghanizadeh 2013 [39]	RCT, placebo-controlled	68 MDD patients, 17-70yo, baseline moderate to severe depression (HDRS-17 ≥ 17)	Fluoxetine (up to) 40mg + lovastatin 30mg	Fluoxetine (up to) 40mg + placebo	6 weeks	HDRS-17
Gougol 2015 [41]	RCT, placebo-controlled	48 MDD patients, 20-70yo, baseline moderate to severe depression (HDRS-17 ≥ 22)	Fluoxetine (up to) 40mg + simvastatin 20mg	Fluoxetine (up to) 40mg + placebo	6 weeks	HDRS-17
Haghighi 2014 [40]	RCT, placebo-controlled	60 MDD patients, 18-50yo, baseline severe depression (HDRS-21 ≥ 25)	Citalopram 40mg + atorvastatin 20mg	Citalopram 40mg + placebo	12 weeks	HDRS-21

CABG = Coronary Artery Bypass Graft; HDRS-17 = Hamilton Depression Rating Scale, 17 items; HDRS-21 = Hamilton Depression Rating Scale, 21 items; MADRS = Montgomery-Åsberg Depression Rating Scale; MDD = Major Depressive Disorder; RCT = Randomized Controlled Trial; TAU = Treatment As Usual (included case management, cognitive behavioural therapy, or pharmacotherapy).

All were published in English, but 4 [39–41, 43] were conducted in Iran and 1 [42] in Australia. They all involved patients with diagnosed major depressive disorder, though 1 [42] included a younger population (i.e. 15–25 years old). Though previous episodes of depression were reported, it is unclear for all trials whether the study population included patients with treatment-resistant depression. Four trials [39–42] were placebo-controlled and used different statins (i.e. rosuvastatin, lovastatin, simvastatin, atorvastatin) in addition to an antidepressant, whereas 1 [43] trial compared 2 statins (i.e. simvastatin against atorvastatin) in the absence of concurrent antidepressant treatment. Follow-ups ranged from 6 to 12 weeks and all primary outcomes were measured on standardized scales for depressive symptoms (i.e. HDRS, MADRS).

### Outcome measures

Outcomes were described quantitatively in Table 2.

**Table 2:** Quantitative outcome measures.

			*Early treatment (8 weeks)*					*Late treatment (12 weeks)*						
Study ID	Treatment arm	Participants randomized *N*	Depressive symptoms *Mean (SD)*	Response *N (%)*	Remission *N (%)*	Acceptability *N (%)*	Tolerability *N (%)*	Safety *N*	Depressive symptoms *Mean (SD)*	Response *N (%)*	Remission *N (%)*	Acceptability *N (%)*	Tolerability *N (%)*	Safety *N*
Abbasi 2015 [43]	Simvastatin	29	4.95 (3.98)	16 (55.17)	-	6 (20.68)	1 (3.45)	5	-	-	-	-	-	-
	Atorvastatin	29	8.56 (6.50)	11 (37.93)	-	6 (20.68)	2 (6.90)	0	-	-	-	-	-	-
Berk 2020 [42]	Rosuvastatin	48	19.10 (10.70)	-	-	-	-	-	17.2 (11.0)	22 (64.70)	7 (20.58)	8 (23.52)	0 (0)	38
	Placebo	42	22.10 (10.60)	-	-	-	-	-	20.4 (12.4)	14 (41.17)	6 (17.64)	8 (23.52)	0 (0)	28
Ghanizadeh 2013 [39]	Lovastatin	34	16.32 (5.03)	-	-	4 (16.66)	1 (4.16)	57	-	-	-	-	-	-
	Placebo	34	20.40 (5.48)	-	-	3 (12.50)	0 (0)	47	-	-	-	-	-	-
Gougol 2015 [42]	Simvastatin	24	6.35 (7.36)	22 (73.33)	14 (46.66)	2 (6.66)	0 (0)	-	-	-	-	-	-	-
	Placebo	24	9.73 (13.22)	14 (46.66)	11 (36.66)	2 (6.66)	0 (0)	-	-	-	-	-	-	-
Haghighi 2014 [40]	Atorvastatin	30	23.47 (3.72)	-	-	0 (0)	0 (0)	-	19.63 (3.16)	1 (2.08)	0 (0)	0 (0)	0 (0)	-
	Placebo	30	25.70 (3.63)	-	-	0 (0)	0 (0)	-	22.03 (3.58)	0 (0%)	0 (0)	0 (0)	0 (0)	-

Depressive symptoms = value on any standardized scale for depressive symptoms; Response = 50% reduction on any standardized scale for depressive symptoms; Remission = depressive score below a pre-specified threshold on any standardized scale for depressive symptoms; Acceptability = number of participants discontinuing treatment (dropouts) due to any cause; Tolerability = number of participants discontinuing treatment (dropouts) due to adverse events; Safety = number of adverse events.

The trials’ primary outcomes mostly focused on changes in the early phase of treatment (i.e. 6–8 weeks), while longer-term outcomes (i.e. 12 weeks) were reported in fewer trials.

In the earlier trial by Ghanizadeh and Hedayati, 68 patients with moderate to severe depression were randomized to 6 weeks of either fluoxetine plus lovastatin or fluoxetine plus placebo: although their depressive scores decreased in both groups, the change was significantly more pronounced in the statin group [39]. A similar trial using simvastatin randomized 48 depressed patients and found comparable results [41]. Another trial with 12 weeks follow-up had a slightly different design: 60 severely depressed patients received citalopram for 1 week and were then randomized to either atorvastatin or placebo adjunction; again, results showed significantly lower depressive scores for the statin group [40]. The most recent article included a larger 15–25 years old sample of depressed patients followed-up for 12 weeks who were randomized to treatment as usual, defined as psychotherapy or antidepressant, plus either rosuvastatin, aspirin, or placebo; in this case, the statin group did slightly better than placebo, but the difference was not statistically significant [42]. Only one trial did not include a placebo arm as it compared simvastatin versus atorvastatin, in absence of concurrent antidepressant treatment, in 58 post-coronary artery bypass graft patients with comorbid mild to moderate depression, showing that depressive scores at 6 weeks decreased more prominently in the simvastatin group [43].

Overall, the 4 placebo-controlled trials [39–42] showed comparable acceptability and tolerability of statins versus placebo, with no significant differences in treatment discontinuation due to any cause or adverse events. In terms of safety, statins were associated with a similar number and type of side-effects compared to placebo as reported in 2 trials [39, 42]. The trial comparing simvastatin to atorvastatin [43] did not show any statistically significant difference in terms of overall adverse events, though patients on simvastatin reported more often dry mouth, loss of appetite, constipation, and daytime drowsiness.

### Data analysis

Forest plots demonstrate the effect sizes with 95% CI for each parameter from each individual trial, as well as pooled results and heterogeneity.

#### Efficacy

**Fig 3:**
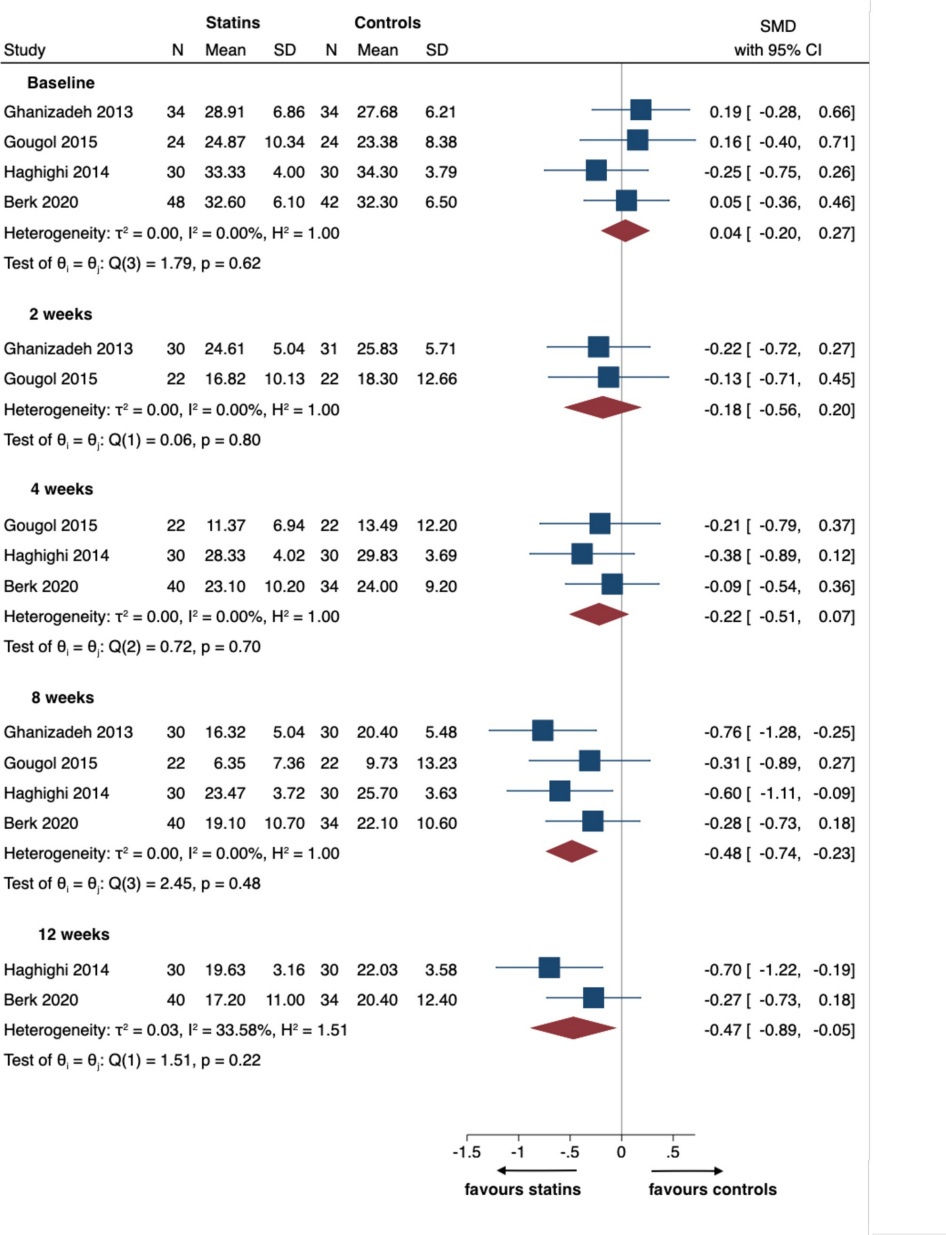
Forest plot for efficacy as mean value for depressive symptoms.

Four trials [39–42] comprising a total of 238 participants were included in the meta-analysis for the primary outcome of efficacy as mean value for depressive symptoms at 8 weeks (Fig 3). Another trial could not be included in this pairwise meta-analysis as it only compared between two statins, without a placebo arm [43].

This showed a moderate effect of statins in improving depressive scores compared to placebo (SMD = -0.48, 95% CI = -0.74 to -0. 22), which was statistically significant. Efficacy at 2 weeks (2 trials [39, 41], 105 participants, SMD = -0.18, 95% CI = -0.57 to 0.20) and at 4 weeks (3 trials [40–42], 178 participants, SMD = -0.22, 95% CI = -0.51 to 0.08) showed a progressive trend towards improvement for the statins’ arm, which however was not statistically significant. Efficacy at 12 weeks (2 trials [40, 42], 134 participants, SMD = -0.47, 95% CI = -0.89 to -0.05) showed a moderate effect of statins in improving depressive scores compared to placebo, which was statistically significant. Calculation of I^2^ did not show any significant heterogeneity for the efficacy outcomes.

Data for response and remission was available for 3 trials [40–42]: 1 [41] for response and remission at 8 weeks, and 2 [40, 42] for response and remission at 12 weeks (forest plots in S1 Fig in S1 File). No studies had measured response and remission at 2 or 4 weeks. Statins were associated with increased response at 8 weeks in only 1 trial [41] (RR = 1.57, 95% CI = 1.10 to 2.25), but remission at 8 weeks and response and remission at 12 weeks did not show any statistically significant difference. Calculation of I^2^ did not show any significant heterogeneity for response and remission.

#### Acceptability, tolerability

**Fig 4:**
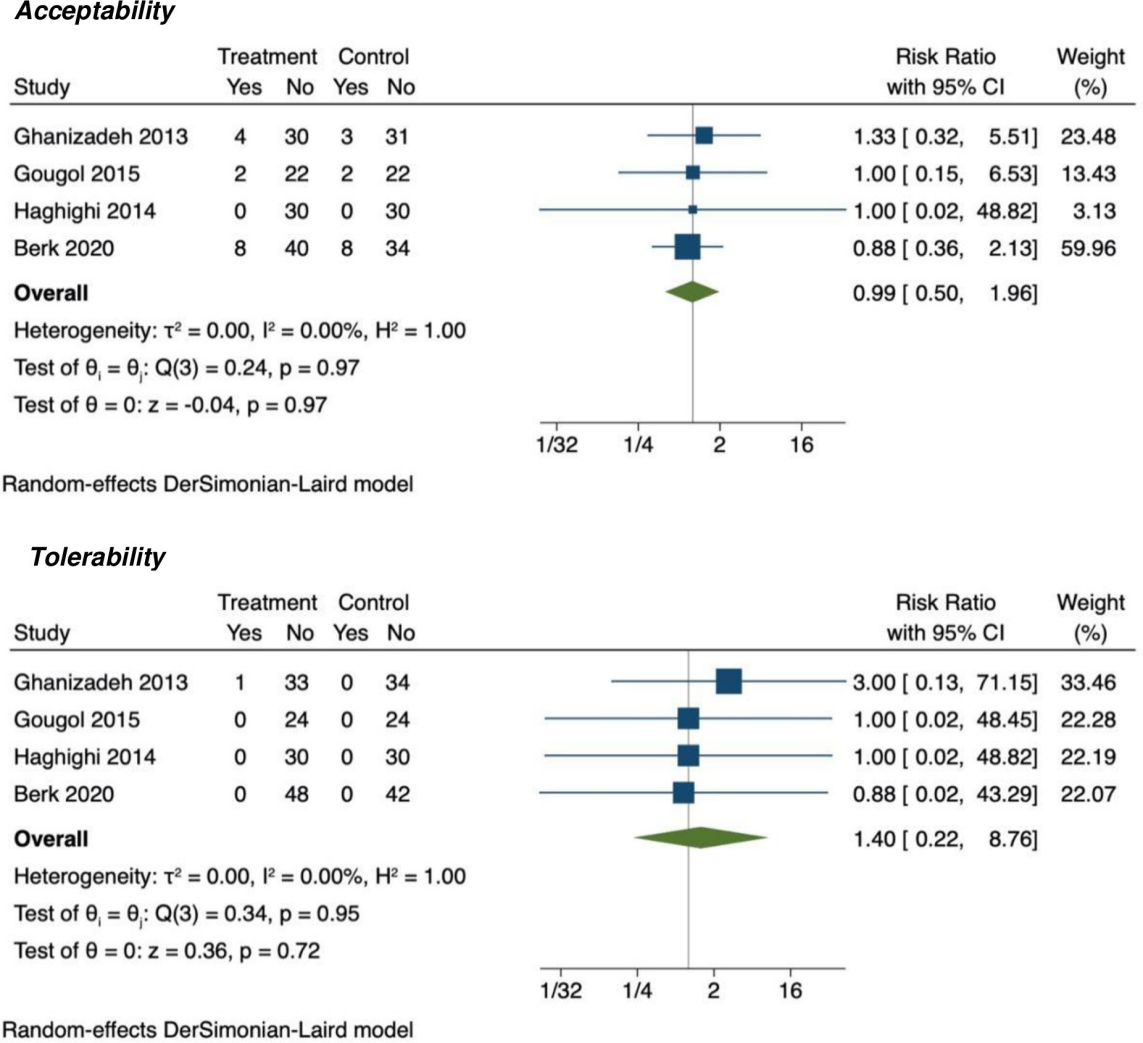
Forest plot for acceptability (number of participants discontinuing treatment [dropouts] due to any cause) and for tolerability (number of participants discontinuing treatment [dropouts] due to adverse events).

Neither acceptability (RR = 0.99, 95% CI = 0.50 to 1.96) nor tolerability (RR = 1.40, 95% CI = 0.22 to 8.76) proved significantly different between statins and placebo, with no evidence of heterogeneity (see Fig 4).

#### Safety

Statins confirmed their safety in this population of depressed patients, with no report of serious adverse events across all trials [39–42]. The most commonly reported side-effects were gastrointestinal (e.g. nausea, vomiting, decreased appetite), pains (e.g. generalized pain, headache, abdominal pain, backpain), psychiatric (e.g. nervousness, insomnia), itches, common cold (S1 Table). Amongst the placebo-controlled trials, only 2 reported the number and type of side-effects separately for statins (specifically, lovastatin and rosuvastatin) and placebo [39, 42]; forest plots of these trials showed that side-effects were equally distributed between the two arms (forest plots in S1 Fig in S1 File). The trial by Gougol et al. did not report detailed data about side-effects, but claimed that no significant difference was found between simvastatin and placebo [41]. The trial by Haghighi et al. reported the pooled number of individual side-effects for patients on atorvastatin and placebo, but no information was available to compare between the two [40].

### Risk of bias (quality) and GRADE (certainty) assessment

The quality assessment was described in the table of bias (see S2 Table in S1 File). The studies at lower risk of bias were the most recent ones [40–43], which were reported in more detail and had a low risk of bias for the crucial items of allocation concealment and blinding of outcome assessment. The older study by Ghanizadeh et al. did not describe any procedure for allocation concealment, therefore it was at unclear risk of bias for allocation concealment and the linked items of blinding of participants, personnel, and outcome assessment [39]. The study by Gougol et al. was at unclear risk of bias for attrition since no reason was given for dropouts and did not describe whether an intention-to-treat analysis was employed [41]. A study protocol had been previously registered for all trials; however, 2 trials had been registered only after recruitment had started [39, 43].

For the most significant outcomes we assessed the certainty of the evidence as reported in the GRADE table (S3 Table in S1 File). Our primary efficacy outcome measure for depressive symptoms at 8 weeks of treatment was downgraded by one level for imprecision due to low sample size, therefore the level of certainty was moderate. Acceptability and tolerability were both downgraded by two levels for imprecision due to low sample size and wide confidence intervals, scoring a low degree of certainty.

Publication bias was not assessed because the number of studies retrieved was below the minimum of 10 trials generally recommended for this analysis [38].

### Are lipophilic statins more efficacious than hydrophilic statins?

An exploratory network meta-analysis was conducted to compare and rank the efficacy of different statins. This analysis allowed the addition of 1 trial [43] that compared simvastatin to atorvastatin but did not have a placebo group. The network plot is shown in the S2 Fig in S1 File.

Analyses are reported in the league table in the S4 Table in S1 File. All statins showed no differences between each other, but only simvastatin (SMD = 0.92, 95% CI = 0.41 to 1.43) and to a lesser extent lovastatin (SMD = 0.77, 95% CI = 0.22 to 1.32) were significantly better than placebo, with a trend towards improvement for atorvastatin and rosuvastatin that did not reach statistical significance. There was no significant network inconsistency χ^2^_(d*f* = 1)_ = 1.09, p = 0.3)

## Discussion

In this systematic review, we comprehensively explored the effects of statins in patients with major depressive disorder over the available clinical trials.

Our meta-analysis showed that statins can be efficacious in addition to antidepressants at 8 and 12 weeks of treatment, with a trend towards improvement seen as early as the second week of treatment. This is in line with previously conducted meta-analyses [30, 32, 34, 35], which however included more heterogenous populations of depressed and non-depressed participants. Compared to a previous meta-analysis on a sample of depressed participants only [33], we could add a more recent trial [42] that lead to a slightly lower effect size for our primary outcome of efficacy at 8 weeks, namely SMD = -0.48 against a previously calculated SMD = -0.73 at 6–12 weeks. We also assessed several other outcomes: apart from an increased response rate at 8 weeks of treatment, statins did not show any statistically significant difference for remission at 8 weeks, response and remission at 12 weeks, and acceptability and tolerability at the studies’ endpoints. Moreover, commonly reported side-effects were similar between statins and placebo. Statins are considered safe drugs: more common side-effect include muscle pain or weakness, elevation of liver transaminases, nasopharyngitis, pharyngo-laryngeal pain, epistaxis, headache, and gastrointestinal disturbances; whereas more serious yet rare adverse events include rhabdomyolysis, new-onset diabetes mellitus, and haemorrhagic stroke [26]. Our latter finding is of interest in confirming the known safety of statin in a psychiatric population affected by depressive disorder; however, it should be noted that adverse events manifesting with long-term use of statins may have not been captured by the relatively short follow-ups of the included studies. Taken together, these results indicate that statins may be a useful treatment option, in addition to an antidepressant, for patients with major depressive disorder. It should be emphasized that all the 4 trials [39–42] included in this meta-analysis had used a statin in addition to an antidepressant strategy. As such, our findings only support the usefulness of adding statins to antidepressants for the treatment of patients with depression, but do not prove that statins alone have an antidepressant’s effect: only a three-arm randomized controlled trial comparing the efficacy on depression scores between a statin alone, an antidepressant alone, and placebo would be able to confirm the latter statement.

We also conducted an exploratory network meta-analysis to investigate whether lipophilic statins are more efficacious in depression compared to hydrophilic ones. This analysis only included few studies, namely 1 trial for rosuvastatin [42] and lovastatin [39] each, and 2 trials for simvastatin [41, 43] and atorvastatin [40, 43] each. Although all the investigated statins proved more efficacious antidepressants than placebo, only the results for simvastatin (largely the most lipophilic statin) and to a lesser extent lovastatin were statistically significant. This effect was mainly driven by an additional trial [43] showing better outcomes for simvastatin over atorvastatin. Taken together, these data might support the hypothesis that a higher degree of lipophilicity is associated with larger effect sizes for efficacy in depression; however, this finding must be considered with caution.

All but one of the included studies had been conducted in Iran. The most recent Australian trial [42] did not show a statistically significant difference in efficacy between statin treatment and placebo, mainly driven by elevated standard deviations, although there was a trend towards benefit with the statin. The sample size calculation of this trial was estimated at 270 participants for a 80% power; however, only 130 participants were randomized, therefore the underpower of this trial may explain why the positive therapeutic trend of statins, in the presence of large standard deviations, did not reach statistical significance. From a methodological perspective, this trial had higher quality according to our risk of bias assessment. However, unlike the other studies reported here, this trial employed a hydrophilic molecule, rosuvastatin. The other statins tested, namely atorvastatin, lovastatin, and simvastatin are more lipophilic (see previous Fig 1) and therefore able to cross the blood-brain barrier relatively easily [28]; therefore, the lower effect size seen for rosuvastatin may be also explained by its lesser capability to enter the central nervous system.

Interestingly, a recent historical cohort study (299,298 participants) showed that simvastatin (i.e. the most lipophilic statin) was associated with a higher number of new-onset diagnoses of depression at 3 years compared to hydrophilic statins (hazard ratio = 1.09, 95%CI = 1.02 to 1.16, p = 0.003). However, a depressogenic effect was not observed when all lipophilic (atorvastatin, lovastatin, simvastatin) statins were compared to those with hydrophilic properties (pravastatin, rosuvastatin) (hazard ratio = 1.05, 95%CI = 1.00 to 1.10, p = 0.078) [44]. The latter finding appears to conflict with the results of our network meta-analysis where simvastatin was one of the more effective statins in increasing the therapeutic effect of antidepressant medications. However, in the trials included in our network meta-analysis, statins had been generally combined with an antidepressant and patients with active depression are likely to present a different neurobiological substrate, such as increased inflammation, to the introduction of a statin. A very large (4,607,990 participants) Swedish national cohort study had indeed shown that simvastatin appears to have the most beneficial effect in terms of reducing the odds of depression (odds ratio = 0.93, 95% CI = 0.89 to 0.97, p = 0.001) amongst all statins (odds ratio = 0.92, 95% CI = 0.89 to 0.96, p< 0.001) [45].

In this context, it is noticeable that the latest trials in depression of large-molecule, specific anti-inflammatory agents [46–48], which do not cross the blood-brain barrier), have produced rather disappointing efficacy outcomes. Because there is evidence for central inflammation in depressed patients [49], it is possible that drugs able to modulate brain inflammatory mechanisms directly may be more therapeutically active. Further clinical trials of statins in depressed patients should therefore consider the specific statins profile when selecting the molecule to be used: a currently running randomised controlled trial chose indeed to use simvastatin in addition to antidepressant [50].

Another important observation is that the rosuvastatin trial involved a younger (15- to 25-year-old) sample. Older populations are more likely to have evidence of inflammation likely related to several factors (e.g. BMI, stress, chronic ailments), a concept known as “inflammaging” [51]. Previous authors have supported the importance of targeting a specific subset of depressed patients with concomitant increased inflammatory markers when using anti-inflammatories for antidepressant purpose [52]; in other words, applying a precision medicine approach to a psychopharmacological treatment. For example, a recently registered clinical trial will be using simvastatin in addition to escitalopram in a selected population of patients with depression and comorbid obesity [53]. In view of this, a sample that is less prone to baseline inflammation because of the young age and that had not been pre-selected for elevated inflammatory markers might see less benefit from using a statin as additional antidepressant treatment. Indeed, the previously mentioned Swedish cohort study did show a stepwise reduction in the odds of developing depression with increasing age for statin-users [45].

Our study has several limitations and strengths. There may be trials that have not been included, though our extensive search strategy should have minimized such risk. The overall sample size for the primary and secondary outcomes was small as we only found 5 articles [39–43] matching our inclusion criteria, and only 4 [39–42] could be included in the meta-analysis; such small number of participants was the main reason why we had to downgrade the certainty of the evidence we found. Although publication bias could not be formally assessed, it is possible that studies showing no effect or negative effects of statins on mood may have not been published. Apart from 1 trial [42] on a younger sample, the study’s populations were homogenous. The length of follow-ups did not go further than 12 weeks, hence the longer-term effects of statins on a depressed population could have been missed. The large majority of the included trials had been conducted in a single country, which may limit the generalizability of our pooled estimates. Two trials [40, 42] involved the measurement of blood lipids whose results, if revealed before the primary analyses on depressive scores, may have interfered with the investigators’ blinding. Future clinical trials may minimise such detection bias by analysing patients’ cholesterol levels only after the administration and scoring of depressive scales. Otherwise, most trials followed appropriate methods and the overall quality of evidence was satisfactory. Our methodology and statistical analysis, as per pre-registered protocol, were robust and followed the recommended practices and guidelines for systematic reviews which, along with low heterogeneity for all outcomes, support the validity of our results.

In conclusion, our study provides evidence suggesting the efficacy (moderate certainty), acceptability, tolerability, and safety (low certainty) of statins in addition to antidepressant treatment for patients with majordepressive disorder. Larger clinical trials in a variety of locations and settings, ensuring that blinding is maintained throughout the study, and potentially preferring the use of simvastatin or other lipophilic statins are needed to test these findings.

## Supporting information

S1 Checklist (DOC)

S1 File (DOCX)

S1 Table Table of side-effects. (XLSX)
