# Supplementary material for: Statins for major depressive disorder: A systematic review and meta-analysis of randomized controlled trials
Source: PLoS One. 2021 Mar 30;16(3):e0249409. doi: 10.1371/journal.pone.0249409 (PMC8009386; doi:10.1371/journal.pone.0249409)
Supplement: S1 File — (DOCX) [file pone.0249409.s002.docx]

# **STATINS FOR MAJOR DEPRESSIVE DISORDER: A SYSTEMATIC REVIEW AND META-ANALYSIS OF RANDOMISED CONTROLLED TRIALS**

**S1 Text– Search algorithm**

(("Hydroxymethylglutaryl-CoA Reductase Inhibitors"[Mesh]) OR *statin OR statins) AND ("Depression"[Mesh] OR "Depressive Disorder"[Mesh] OR "Depressive Disorder, Treatment-Resistant"[Mesh] OR "Depressive Disorder, Major"[Mesh] OR "Sleep"[Mesh] OR "Sleep Wake Disorders"[Mesh] OR "Sleep Initiation and Maintenance Disorders"[Mesh] OR "Sleep Stages"[Mesh] OR "Sleep, REM"[Mesh] OR "Sleep Disorders, Circadian Rhythm"[Mesh] OR "Anhedonia"[Mesh] OR "Anxiety"[Mesh] OR "Anxiety Disorders"[Mesh] OR "Psychomotor Disorders"[Mesh] OR depression OR depressive OR sleep OR insomnia OR sleep disorder OR anhedonia OR anxiety OR psychomotor retardation OR psychomotor impairment OR anx* OR antidepress*))

**S1 Fig– Additional tables and forest plots**

***Remission***

*Remission, early*

**
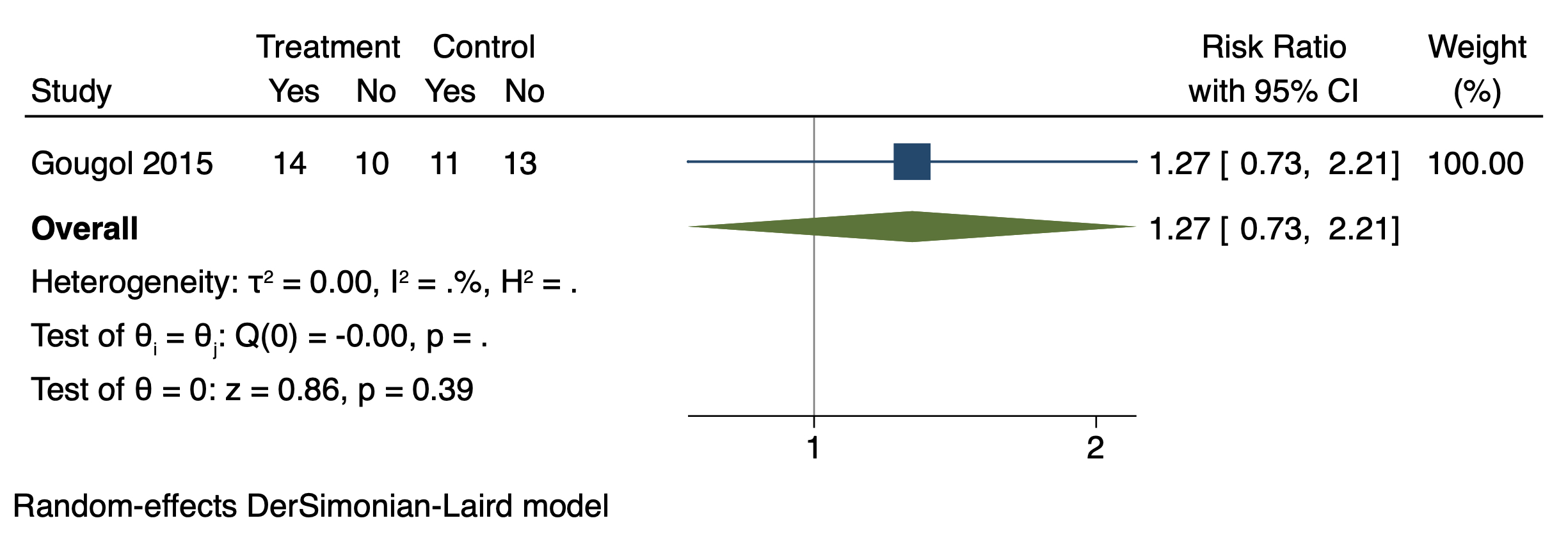
**

*Remission, late*

**
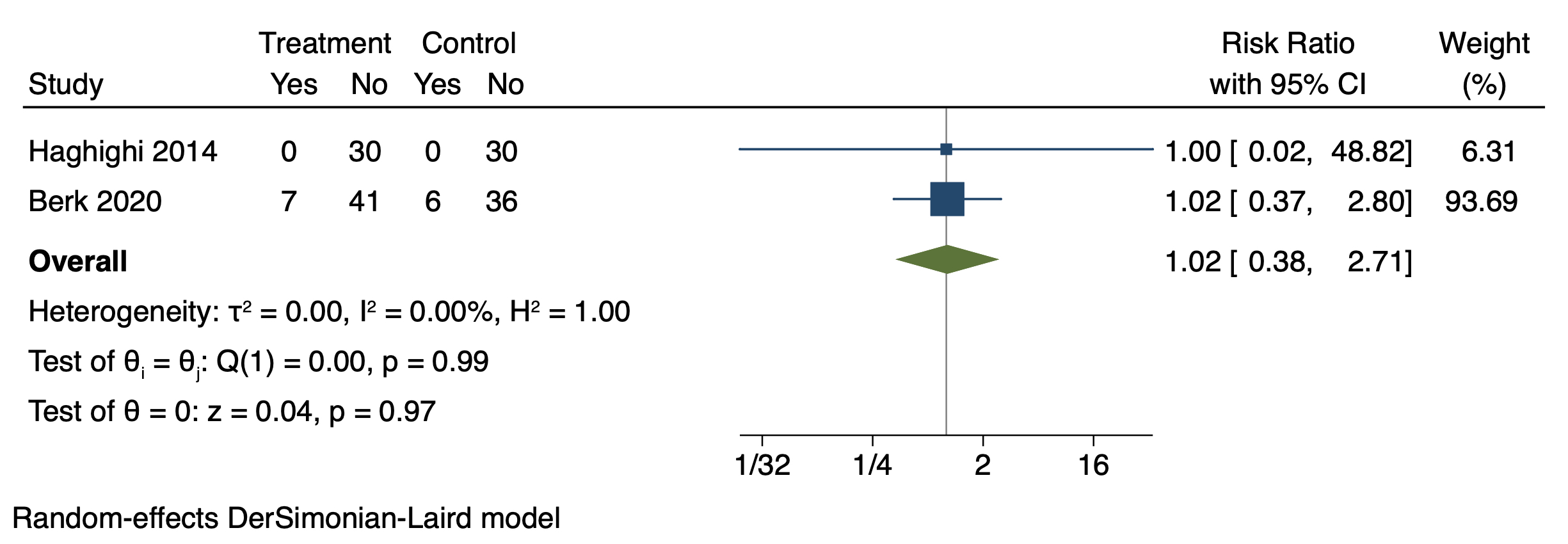
**

***Response***

*Response, early*

**
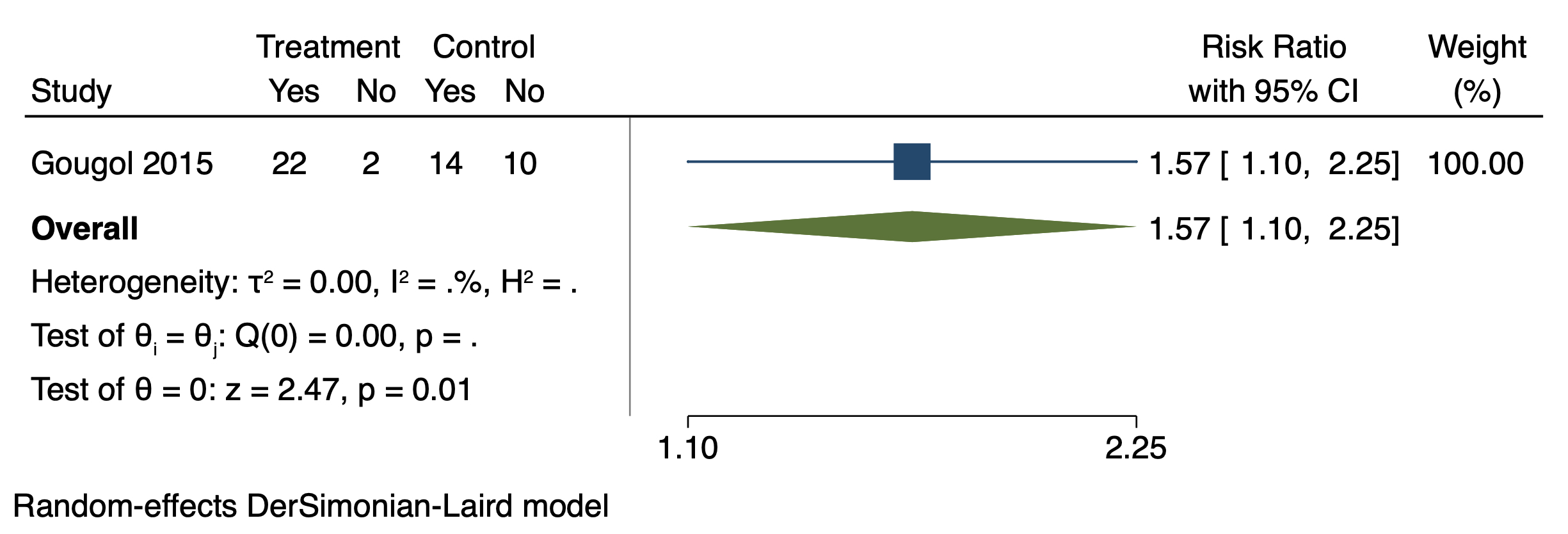
**

*Response, late*

**
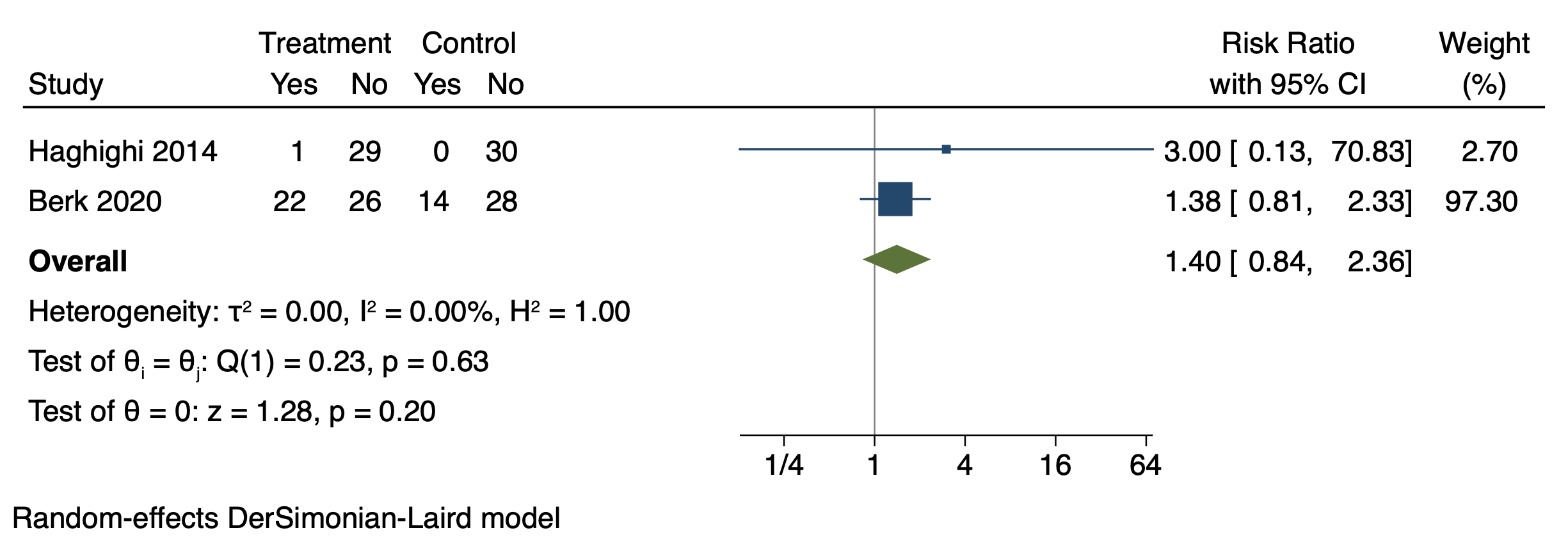
**

***Side-effects***

*Side-effects, Abdominal pain*

**
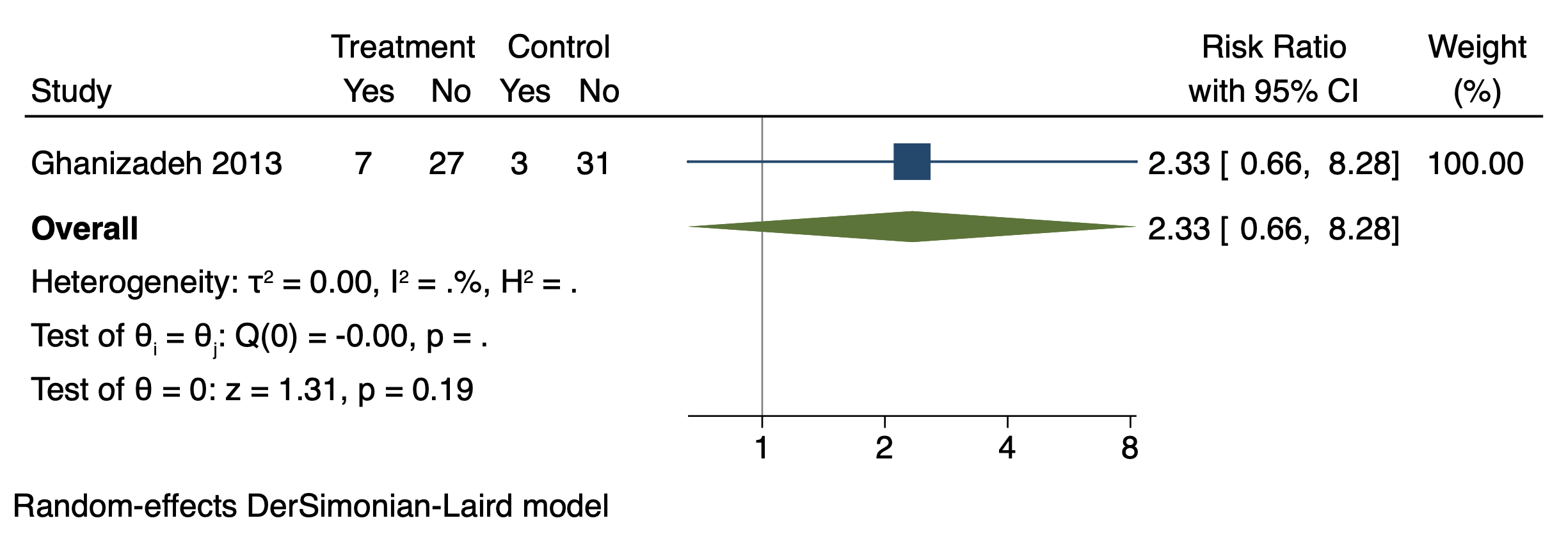
**

*Side-effects, Anxiety*

**
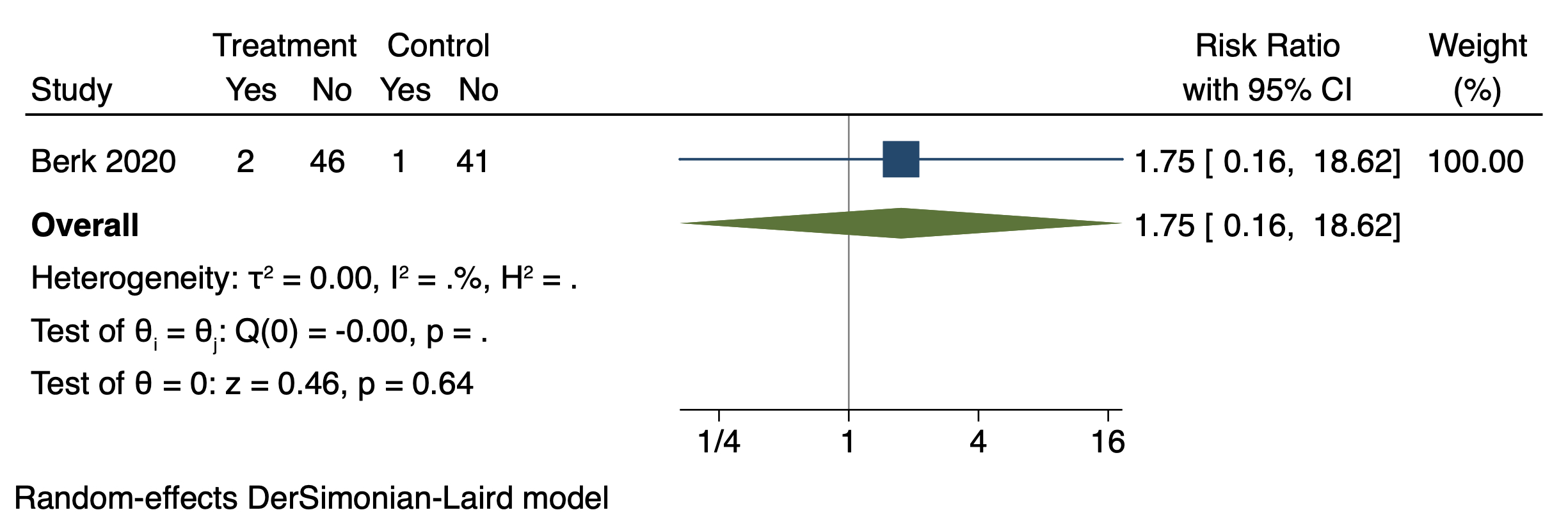
**

*Side-effects, Bleeding*

**
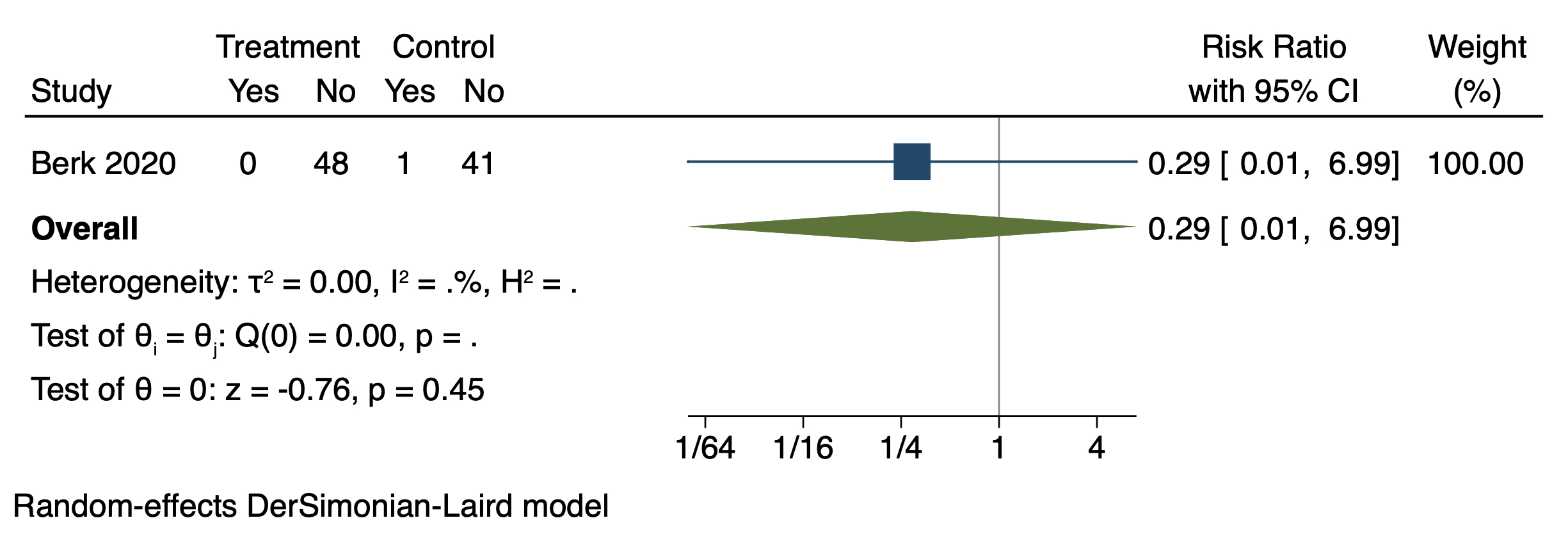
**

*Side-effects, Bruising*

**
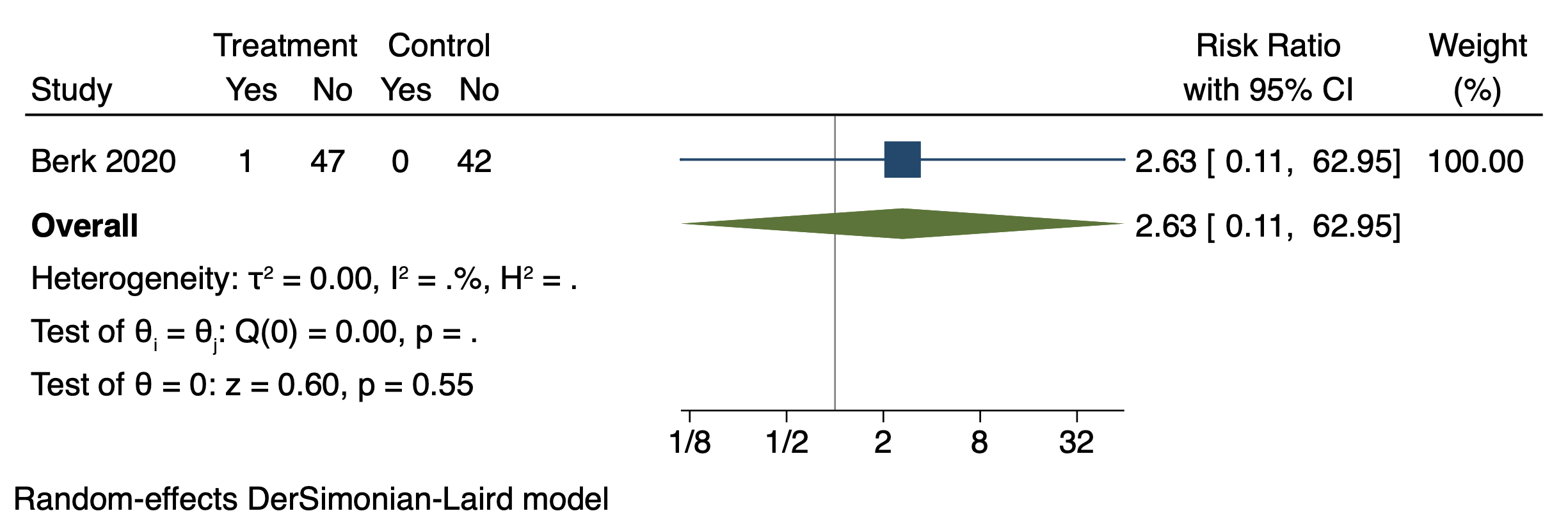
**

*Side-effects, Bruxism*

**
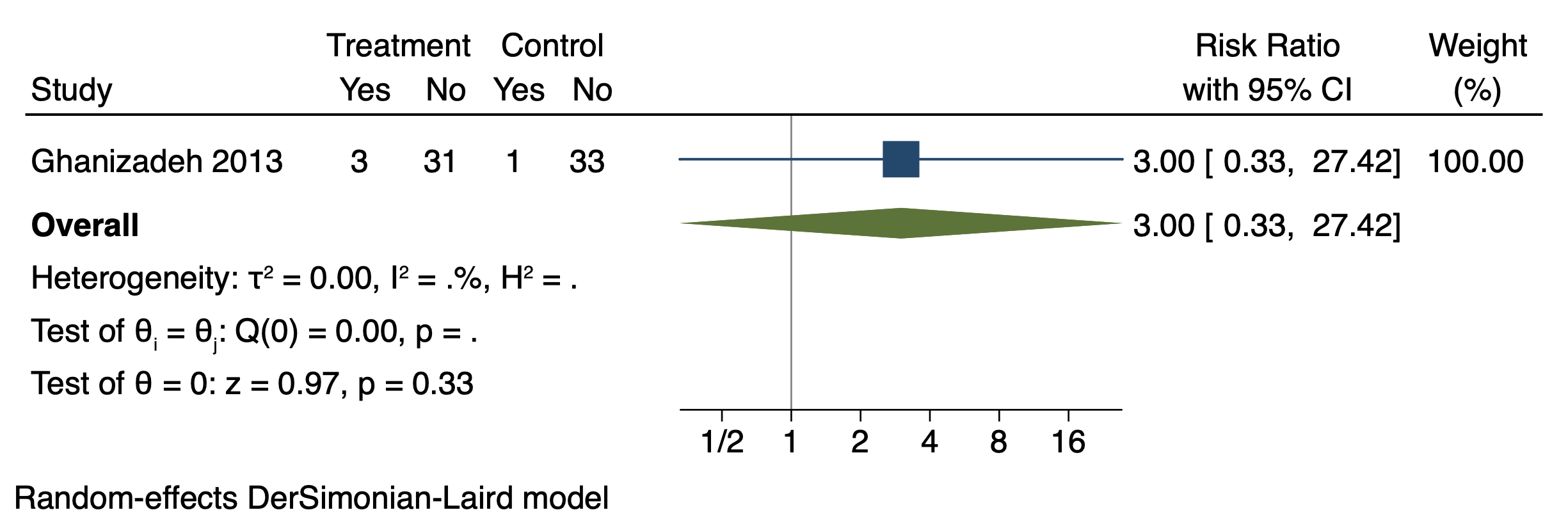
**

*Side-effects, Common cold*

**
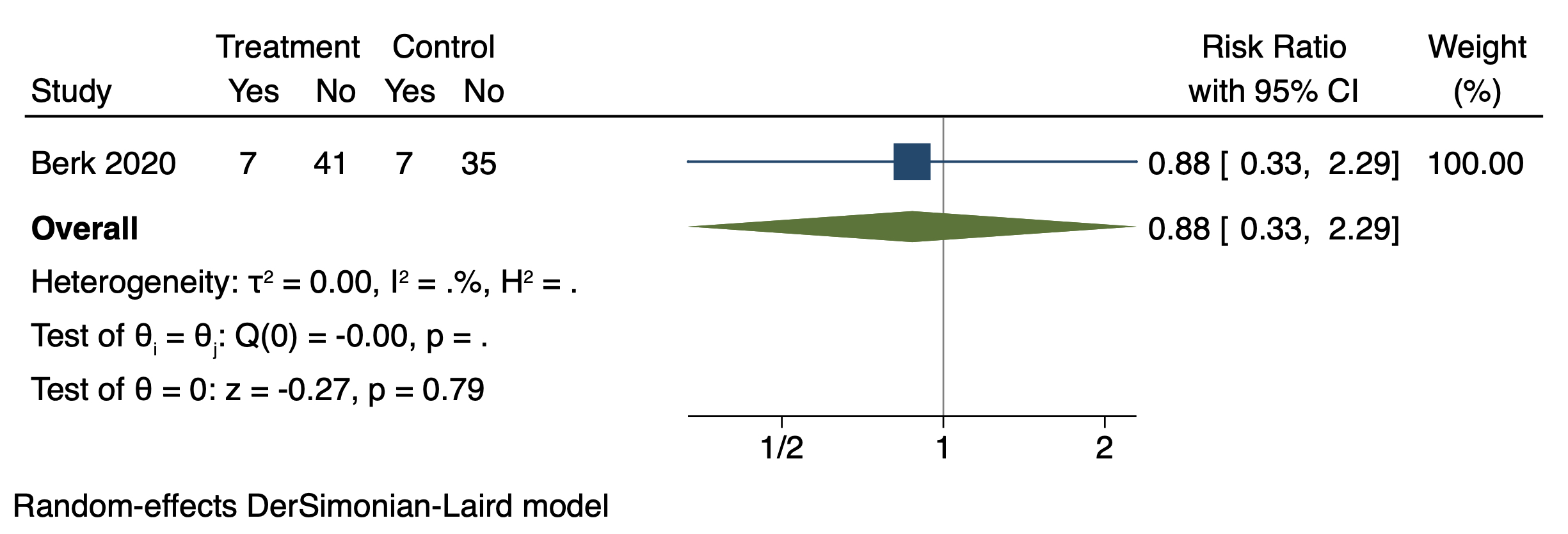
**

*Side-effects, Constipation*

**
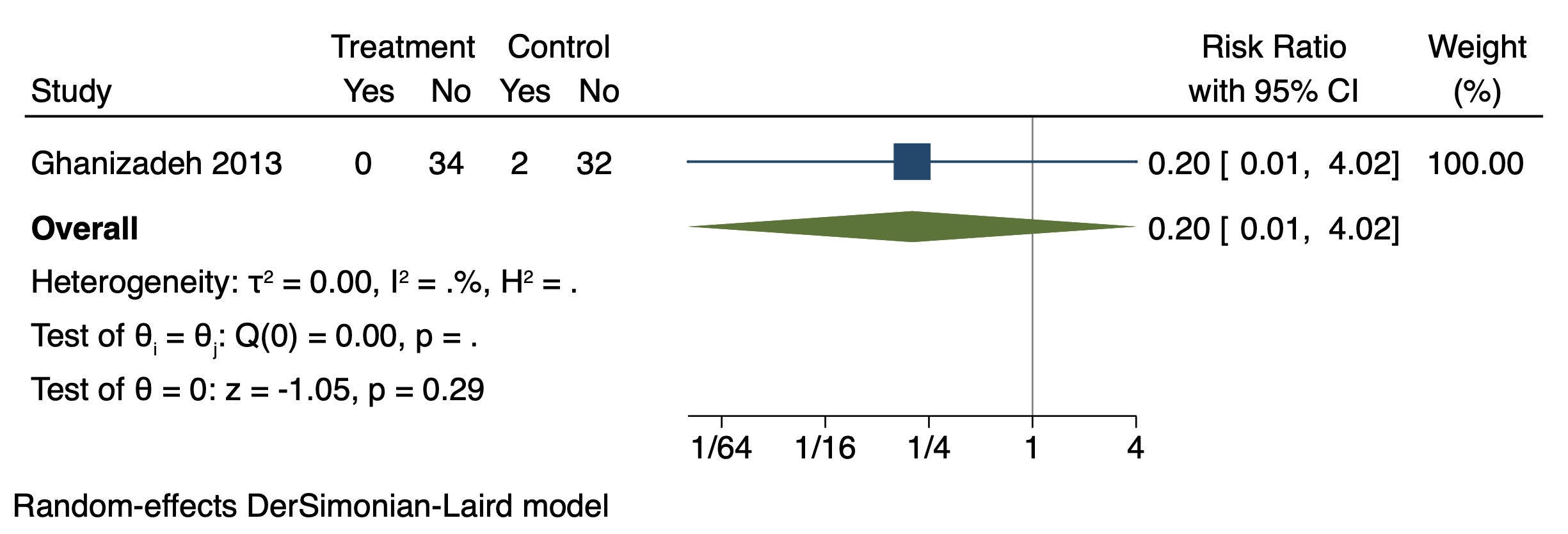
**

*Side-effects, Cough*

**
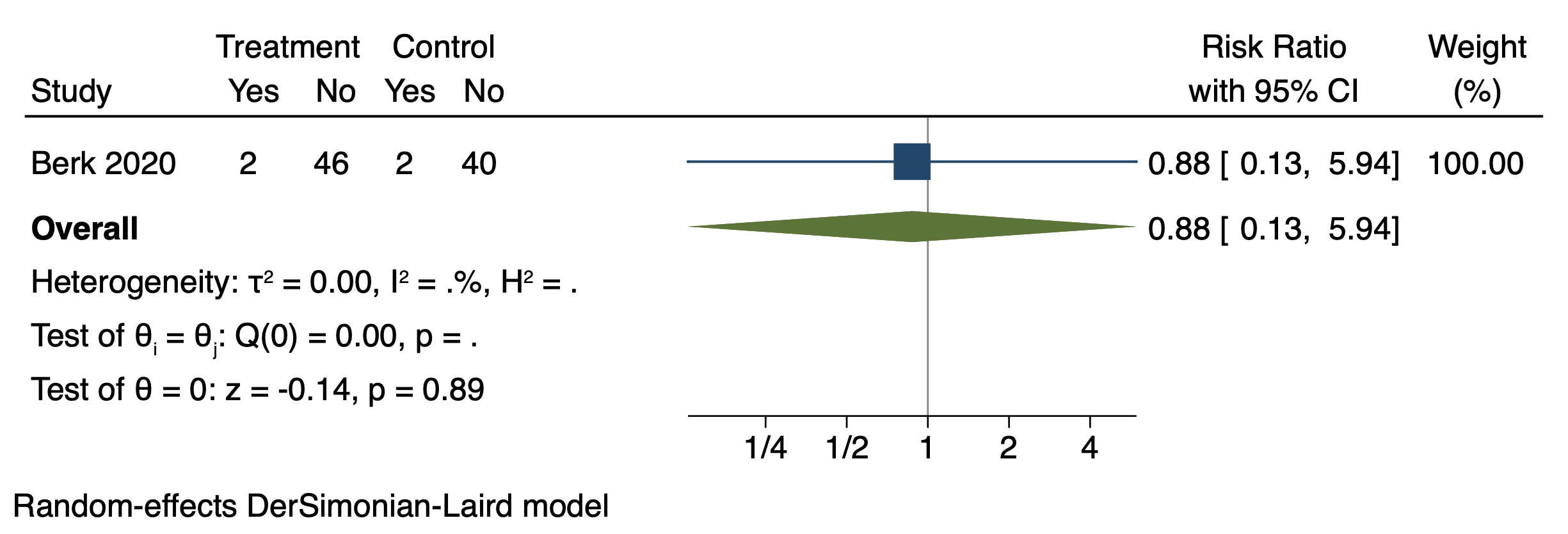
**

*Side-effects, Day-time drowsiness*

**
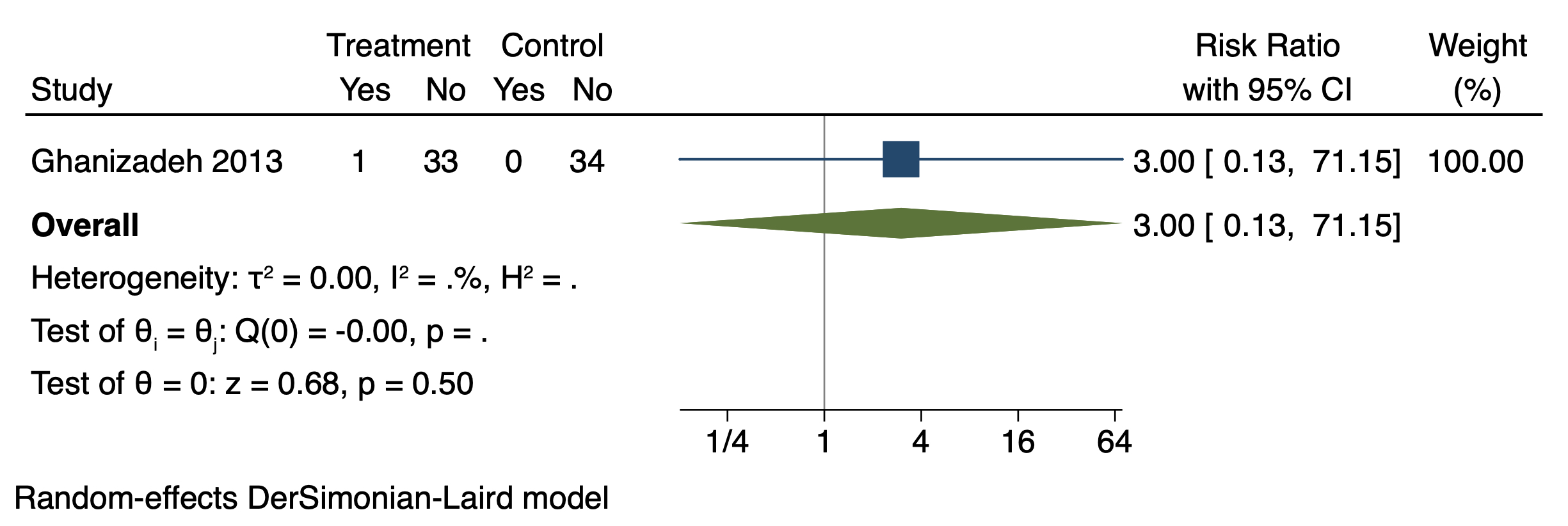
**

*Side-effects, Decreased appetite*

**
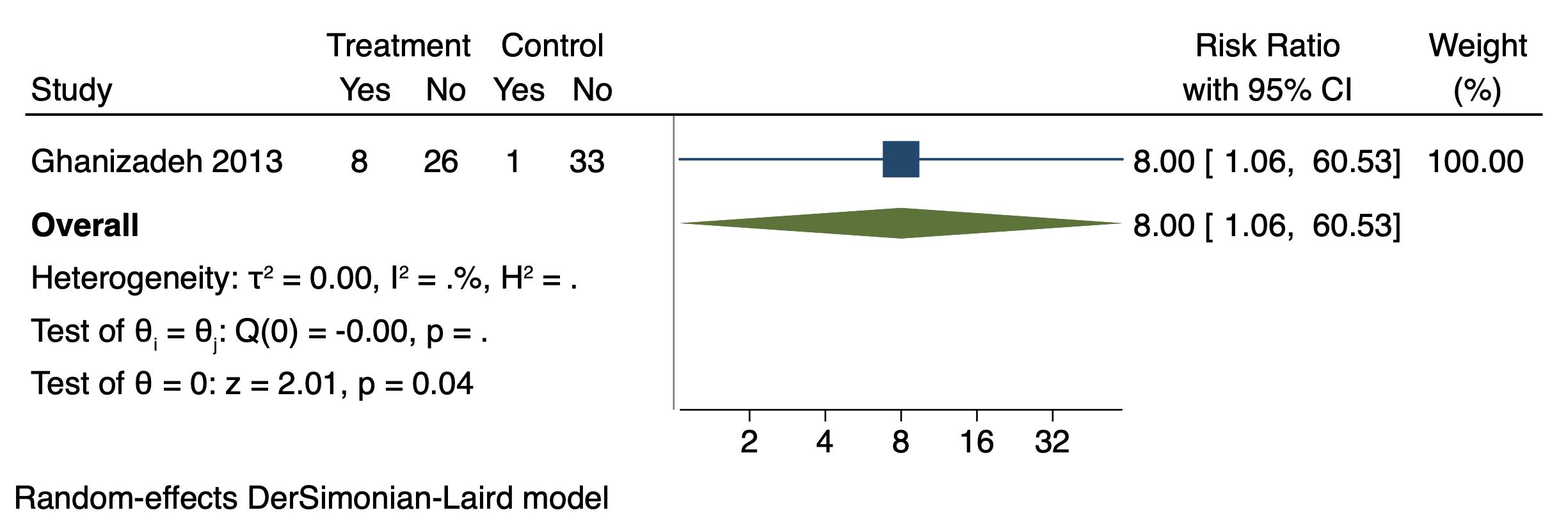
**

*Side-effects, Diarrhea*

**
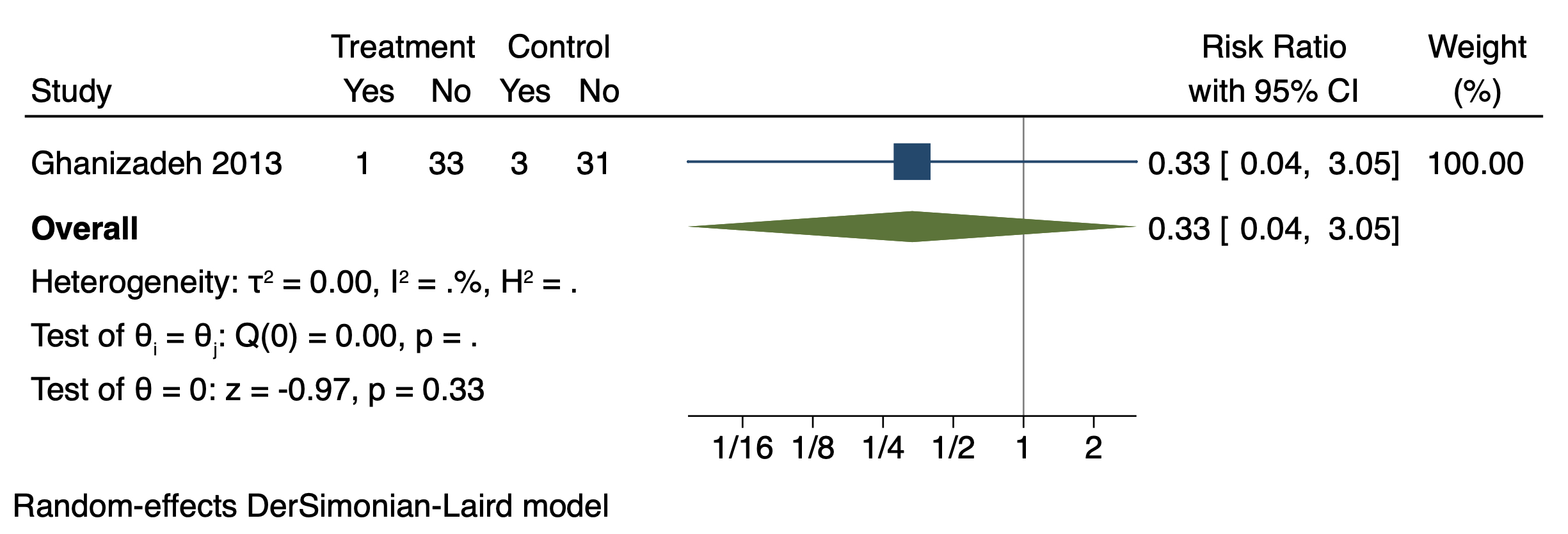
**

*Side-effects, Fainting*

**
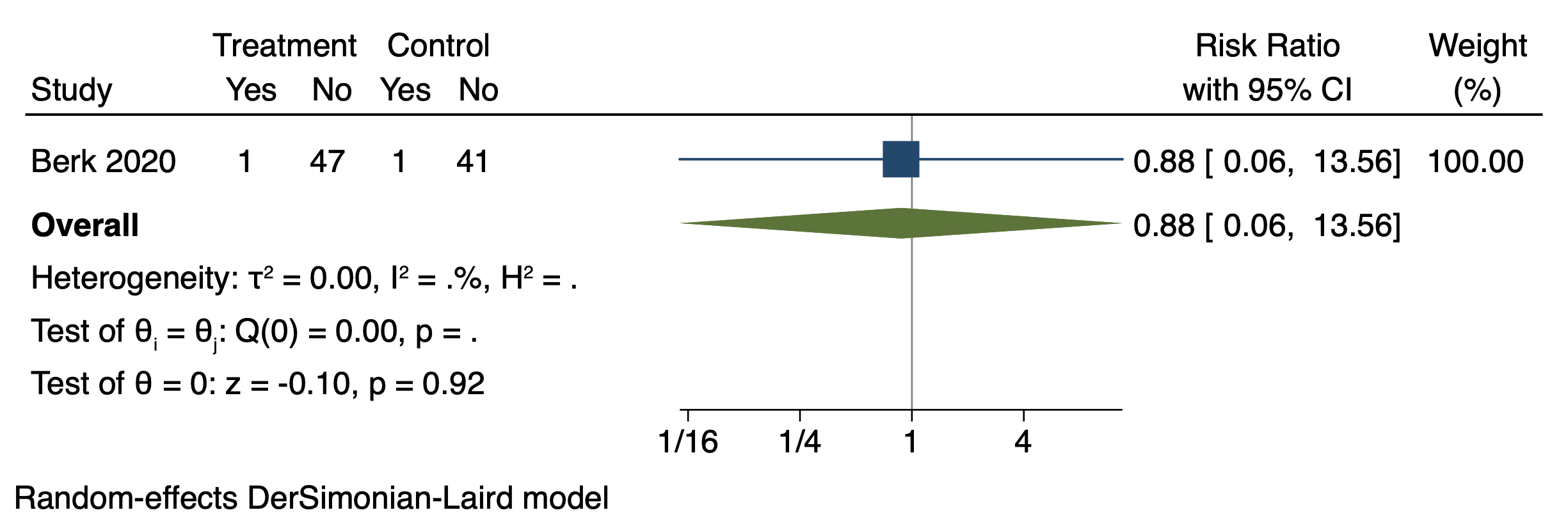
**

*Side-effects, Fatigue*

**
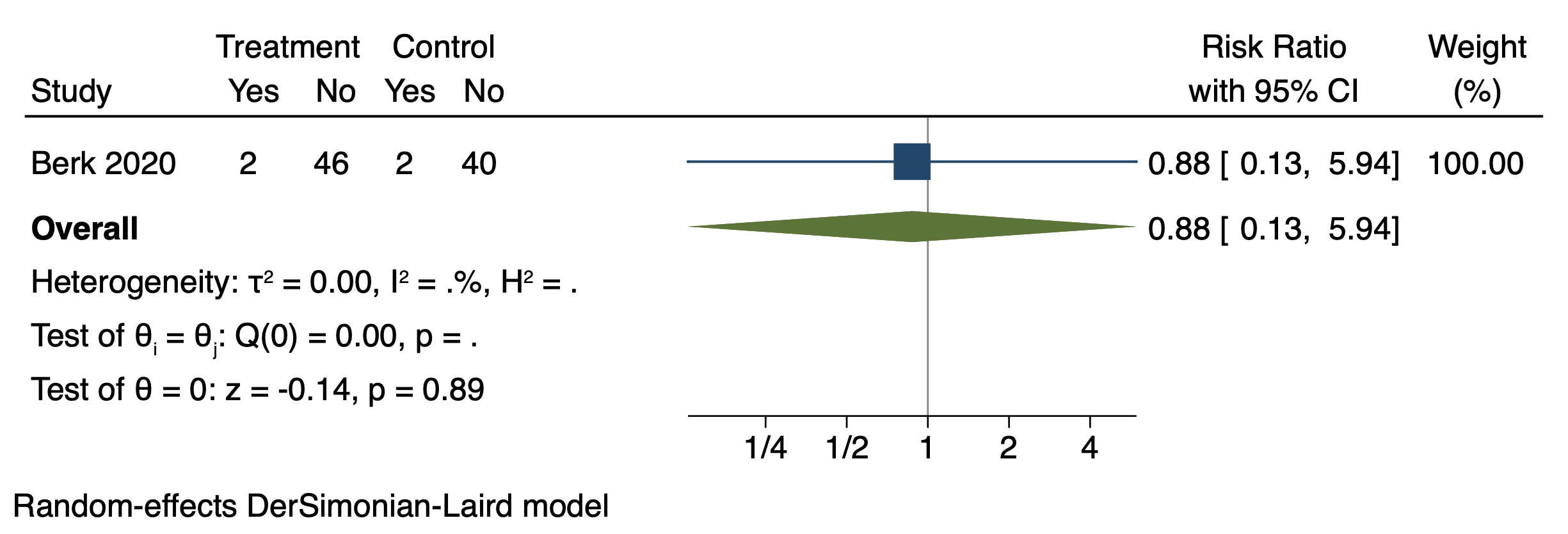
**

*Side-effects, Fever*

**
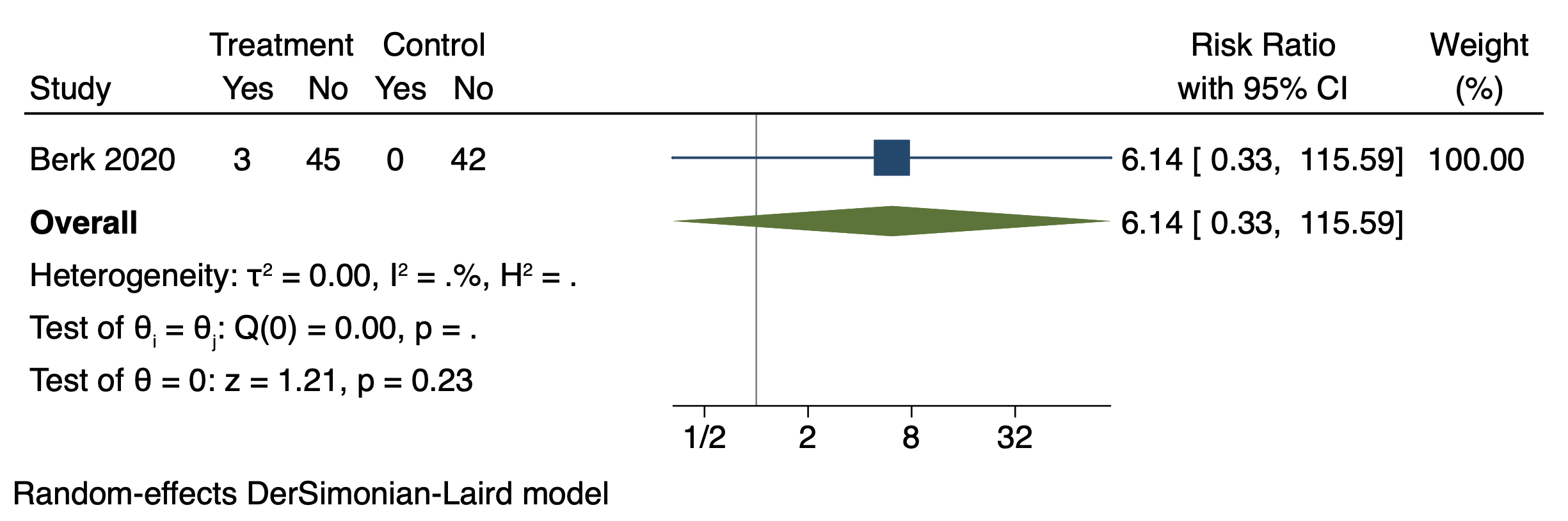
**

*Side-effects, Flu-like syndrome*

**
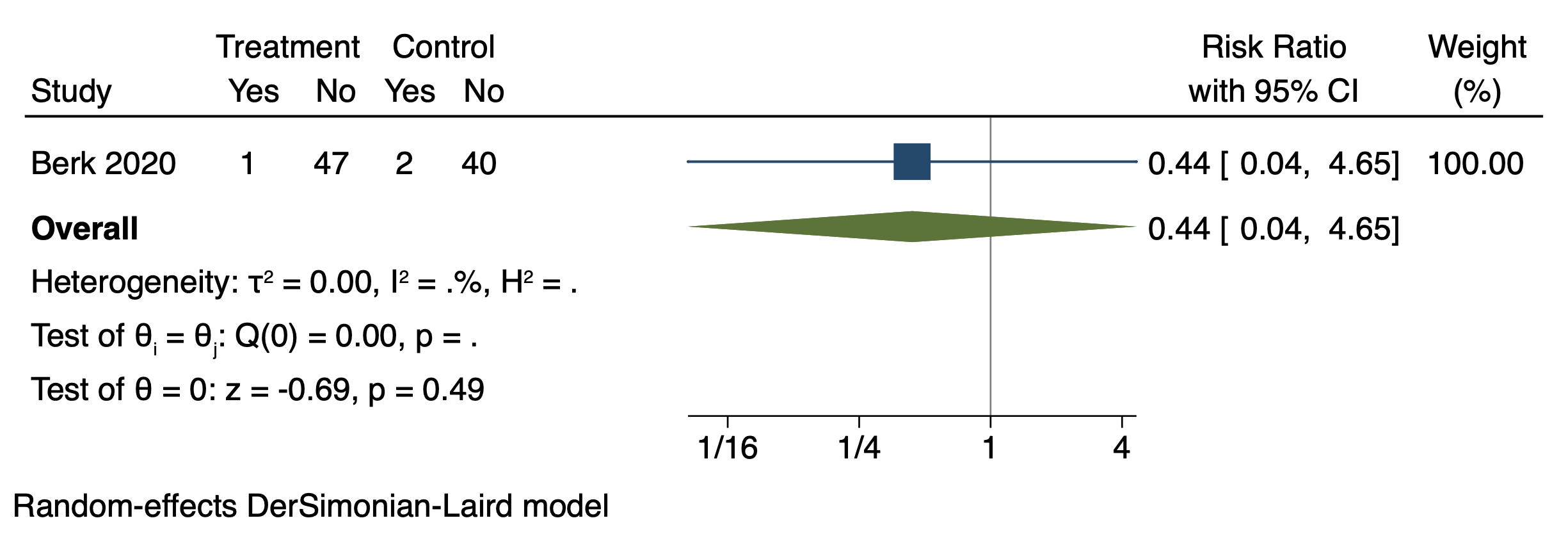
**

*Side-effects, Headaches*

**
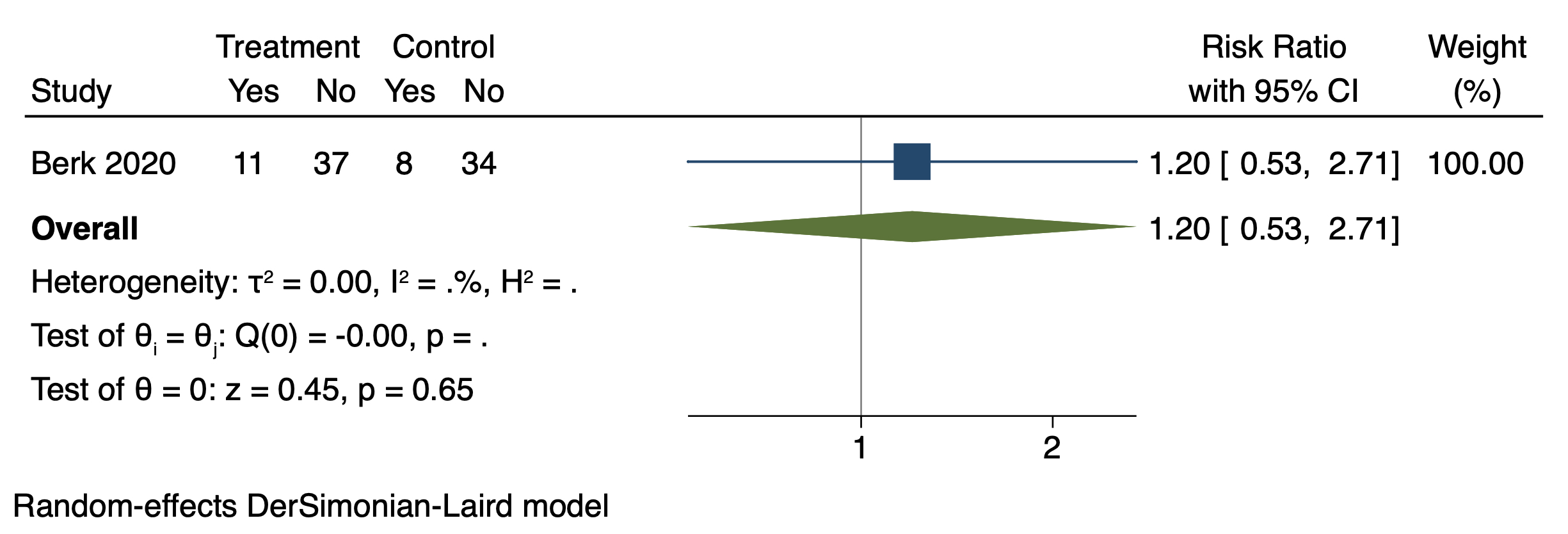
**

*Side-effects, Hospitalization*

**
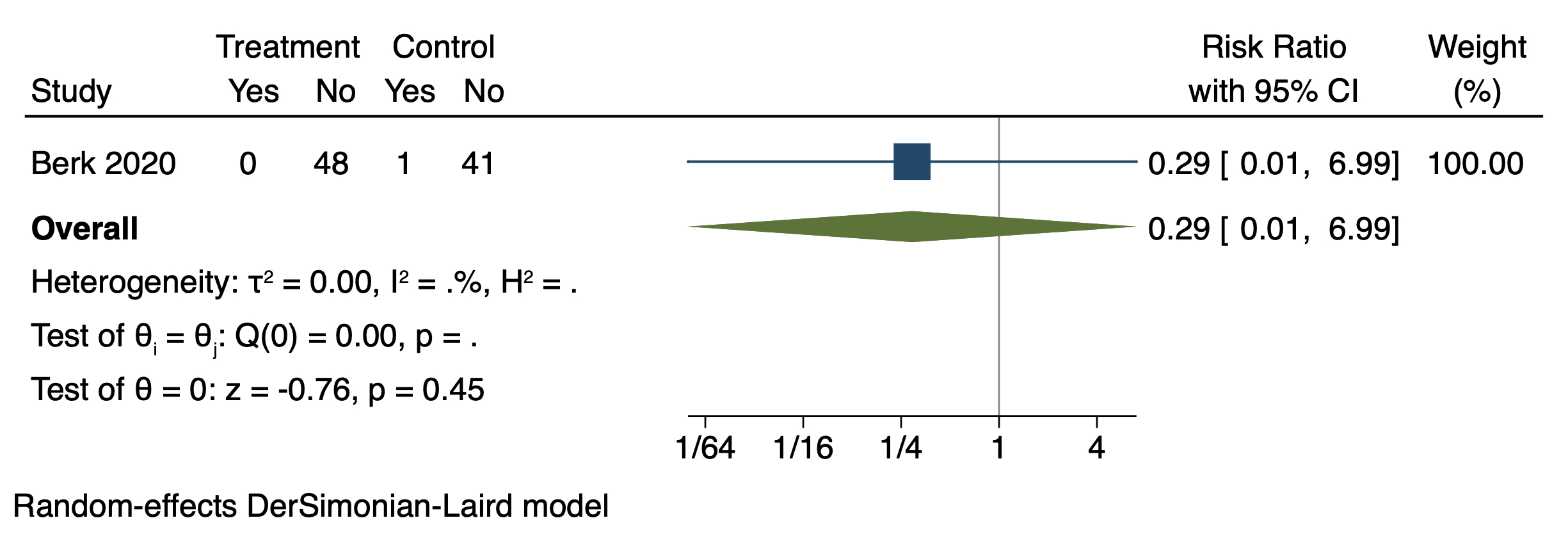
**

*Side-effects, Insomnia*

**
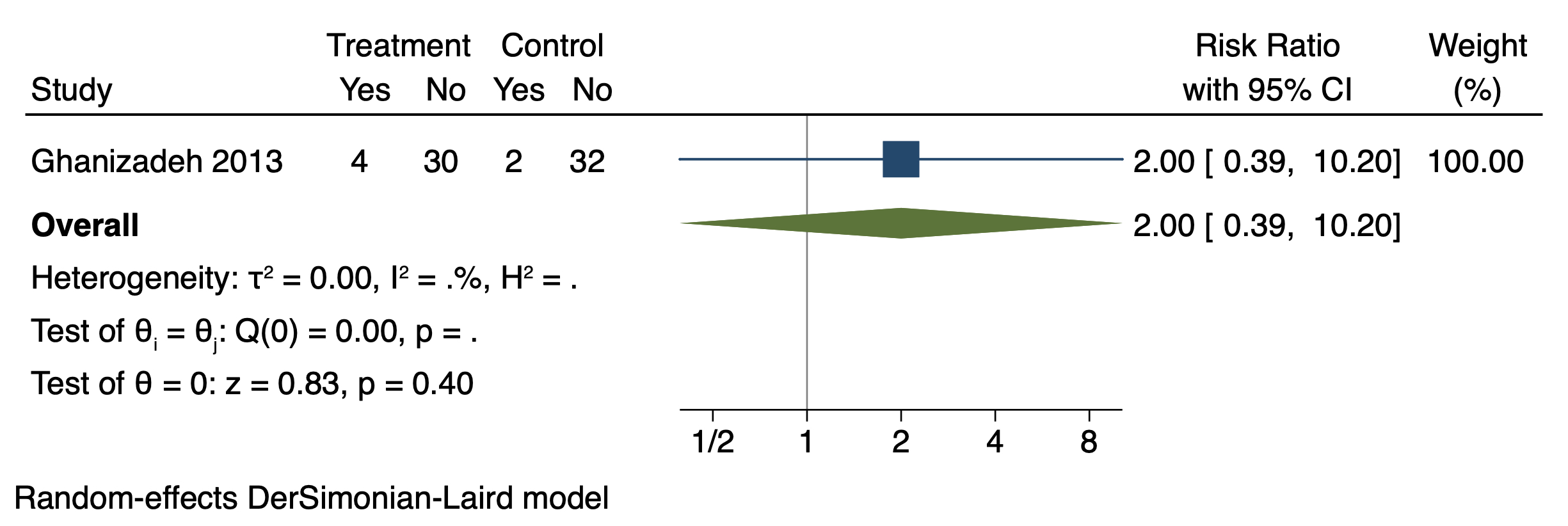
**

*Side-effects, Itches*

**
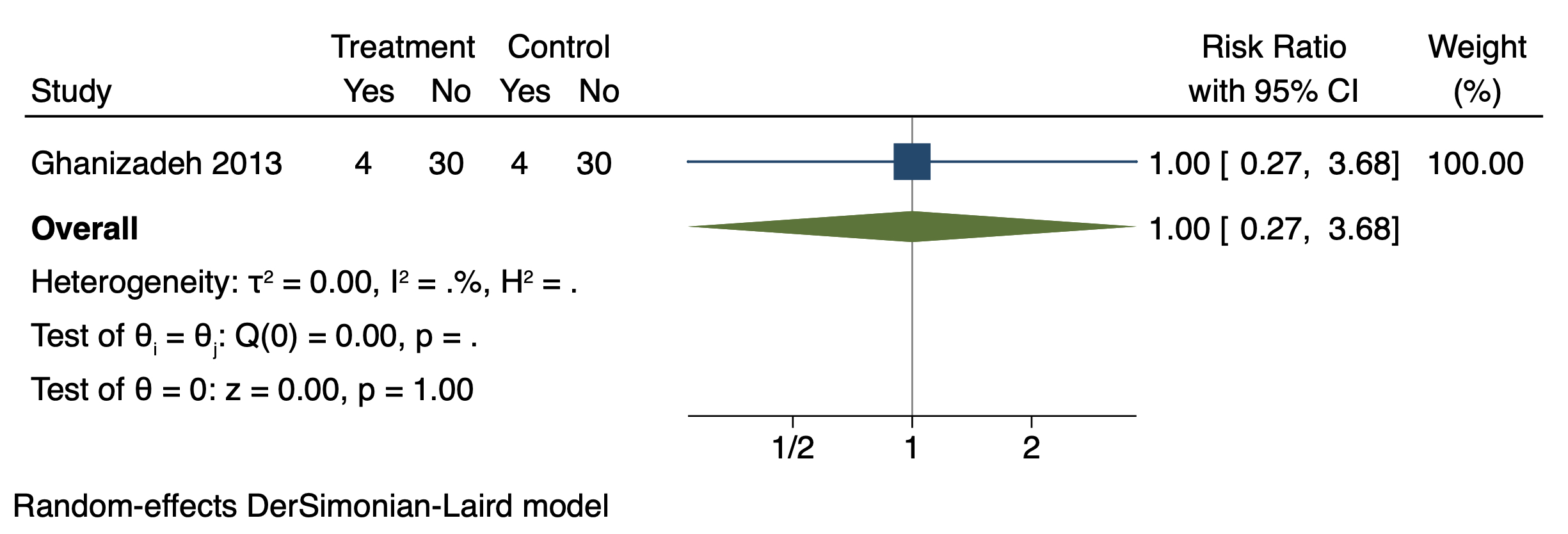
**

*Side-effects, Nausea*

**
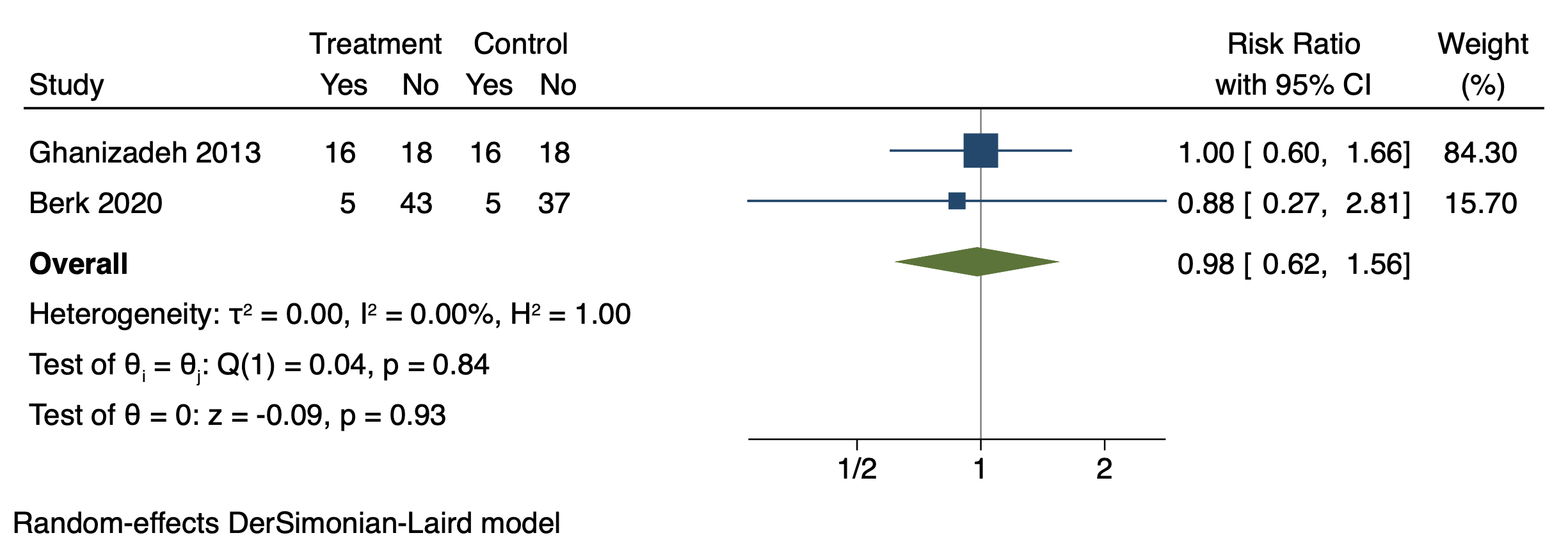
**

*Side-effects, Nervousness*

**
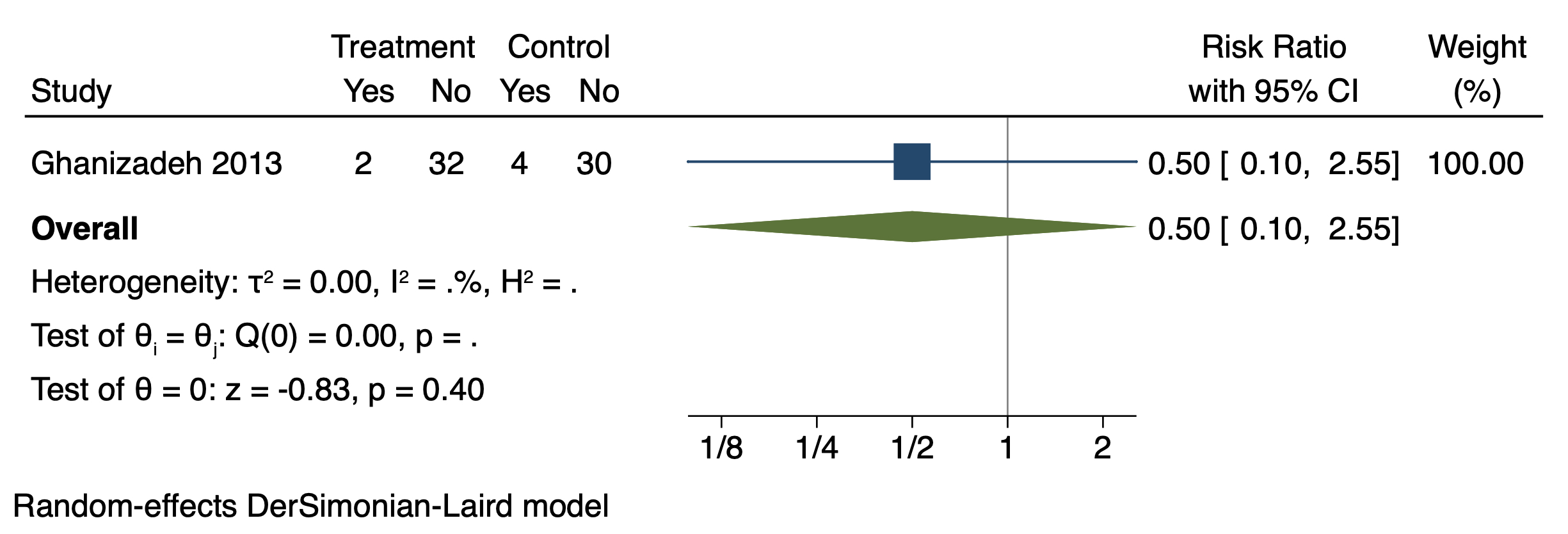
**

*Side-effects, Nightmares*

**
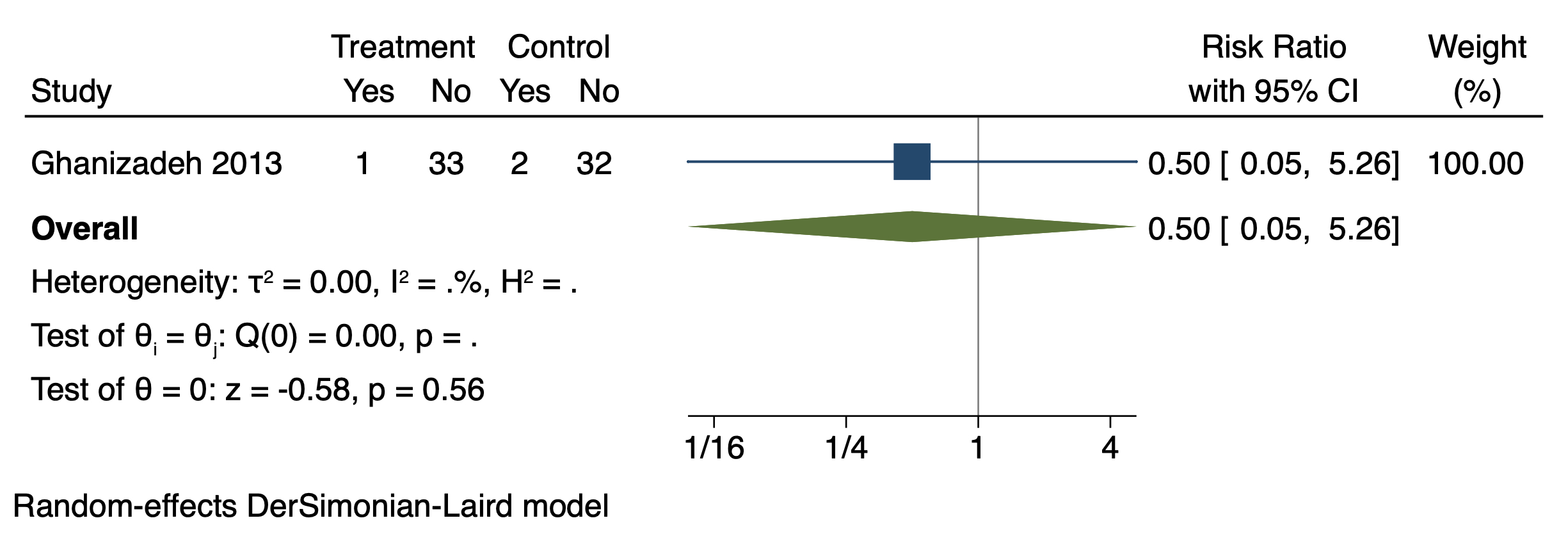
**

*Side-effects, Overdose*

**
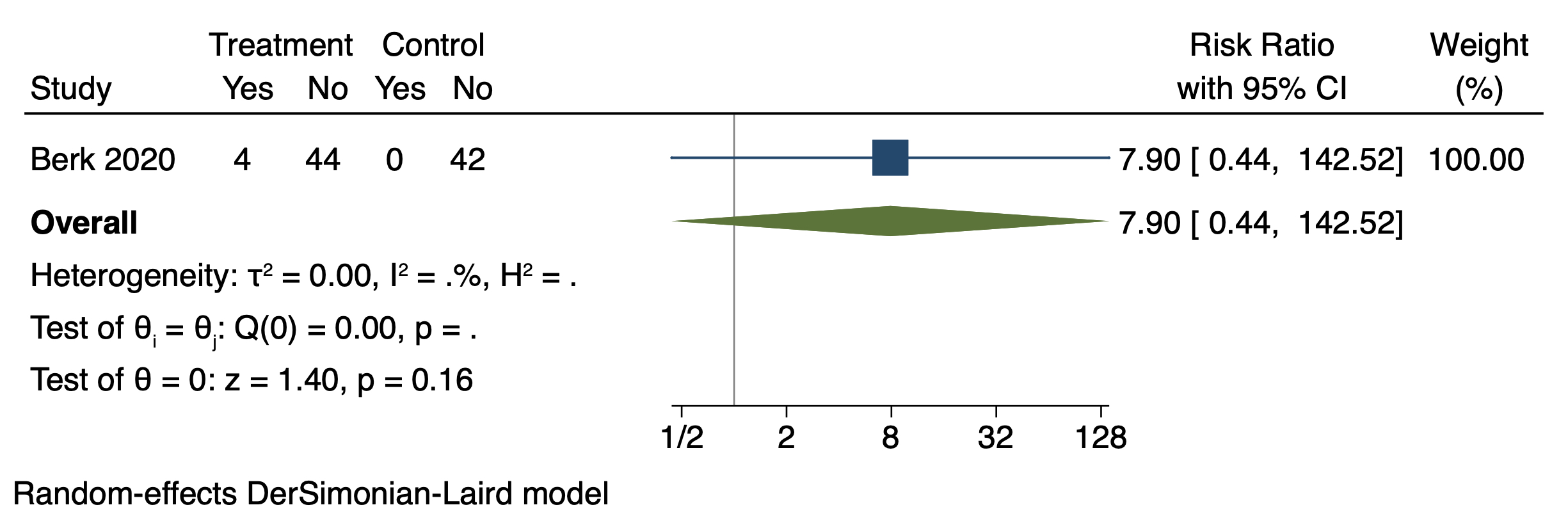
**

*Side-effects, Pain*

**
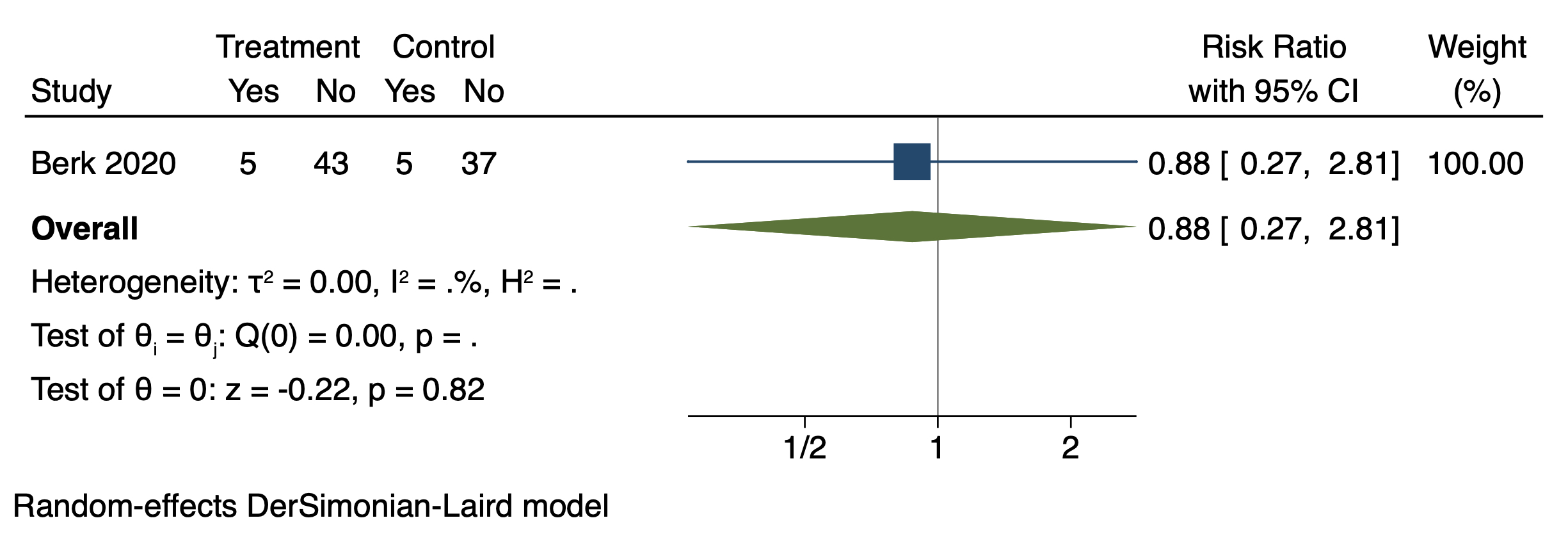
**

*Side-effects, Restlessness*

**
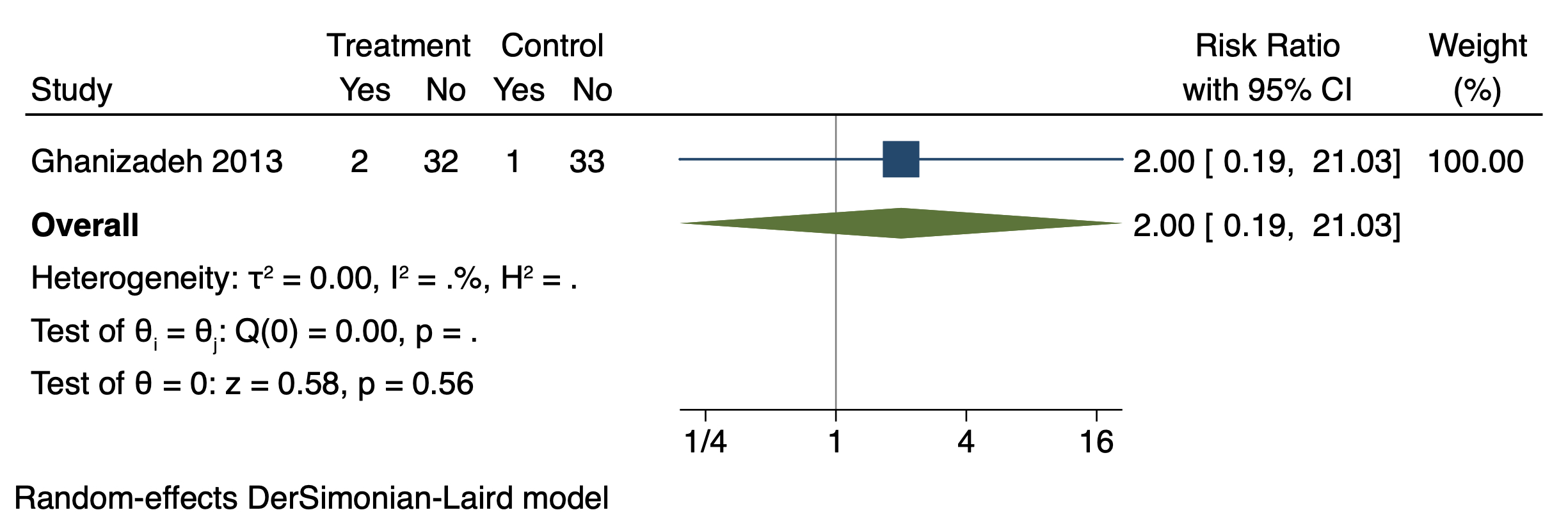
**

*Side-effects, Self-harm*

**
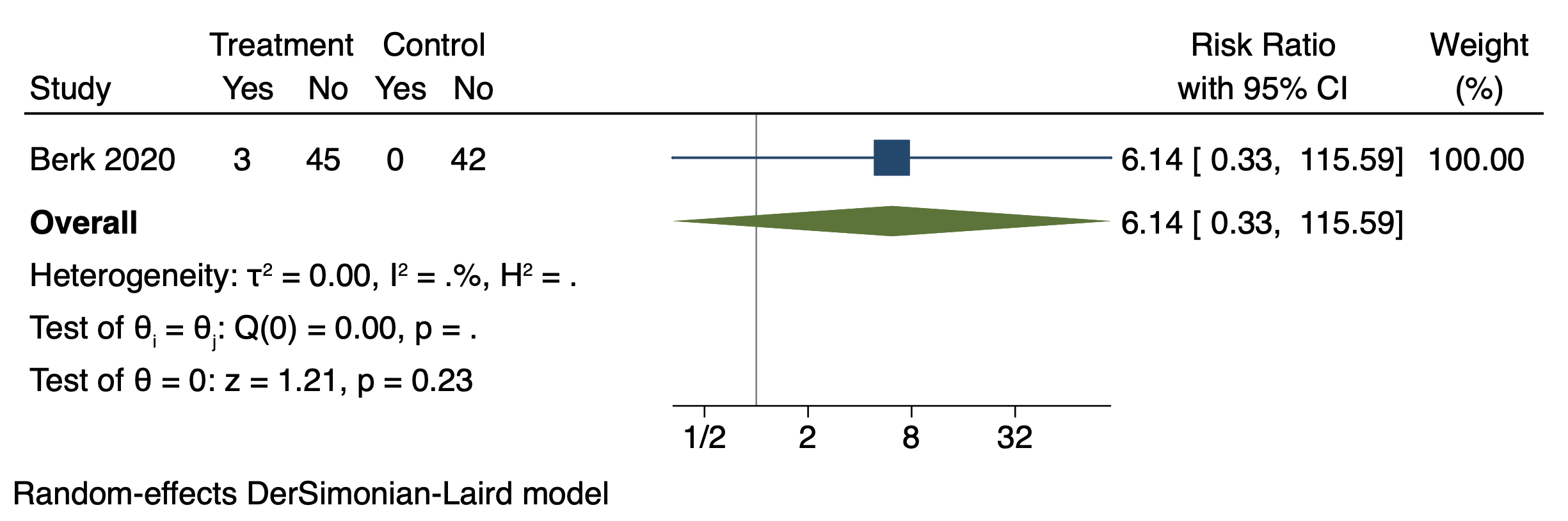
**

*Side-effects, Skin rash*

**
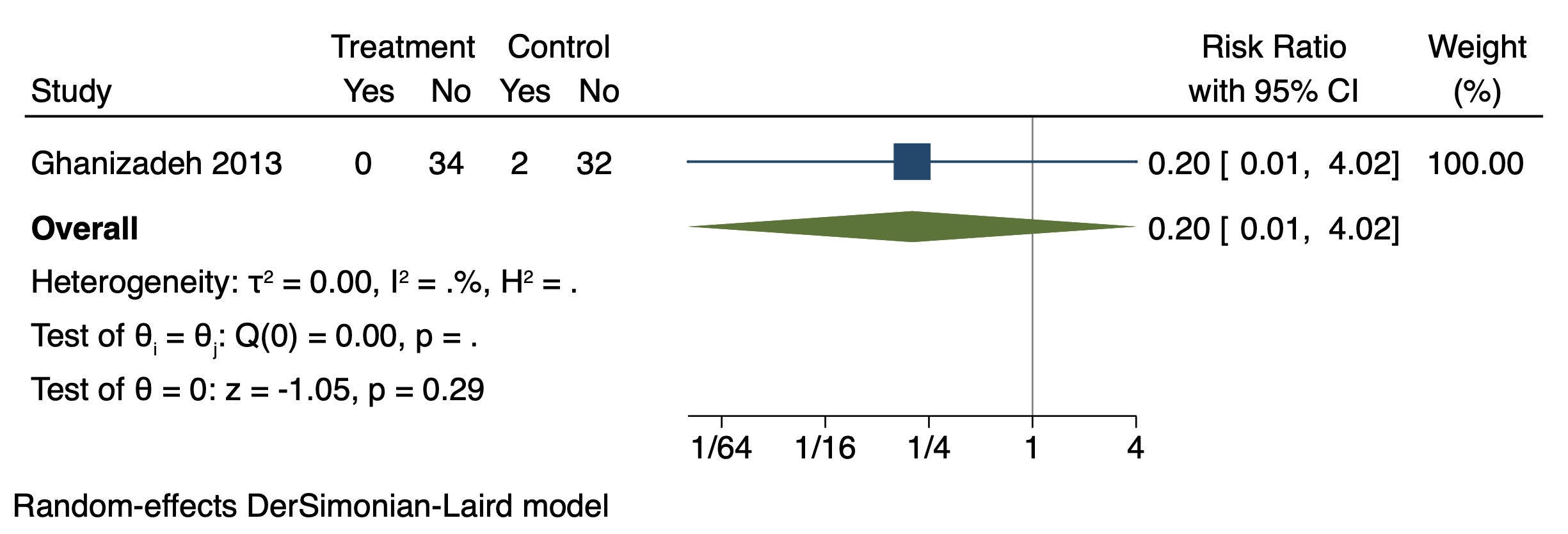
**

*Side-effects, Suicide attempt*

**
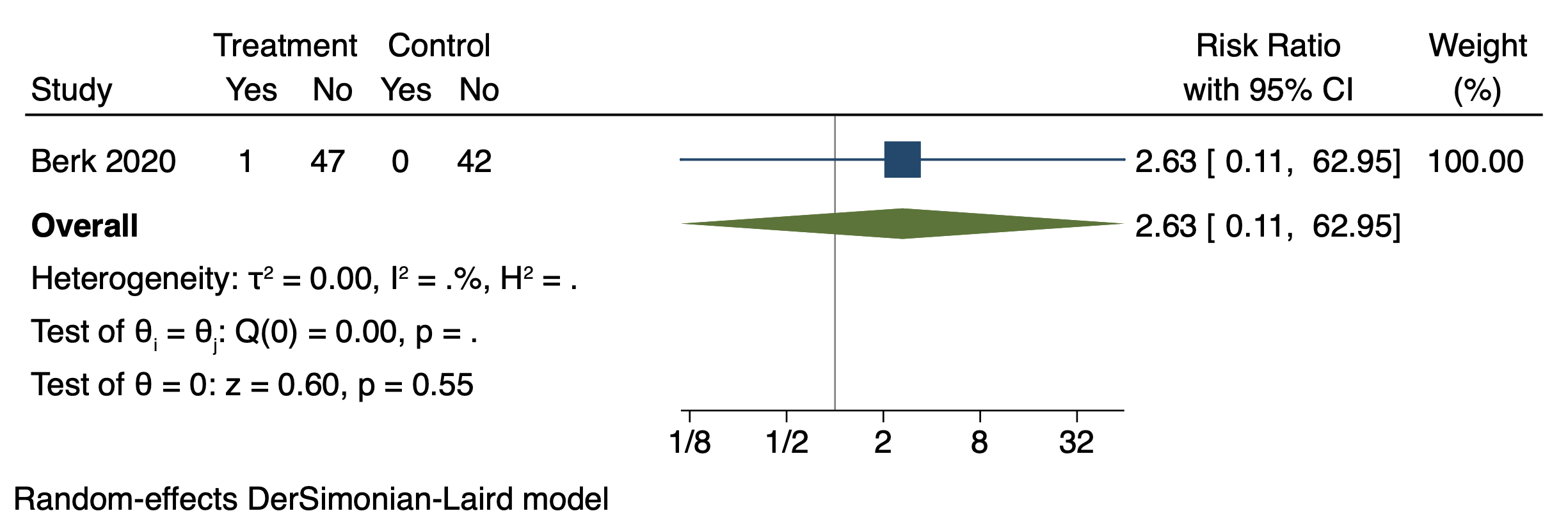
**

*Side-effects, Suicide ideation*

**
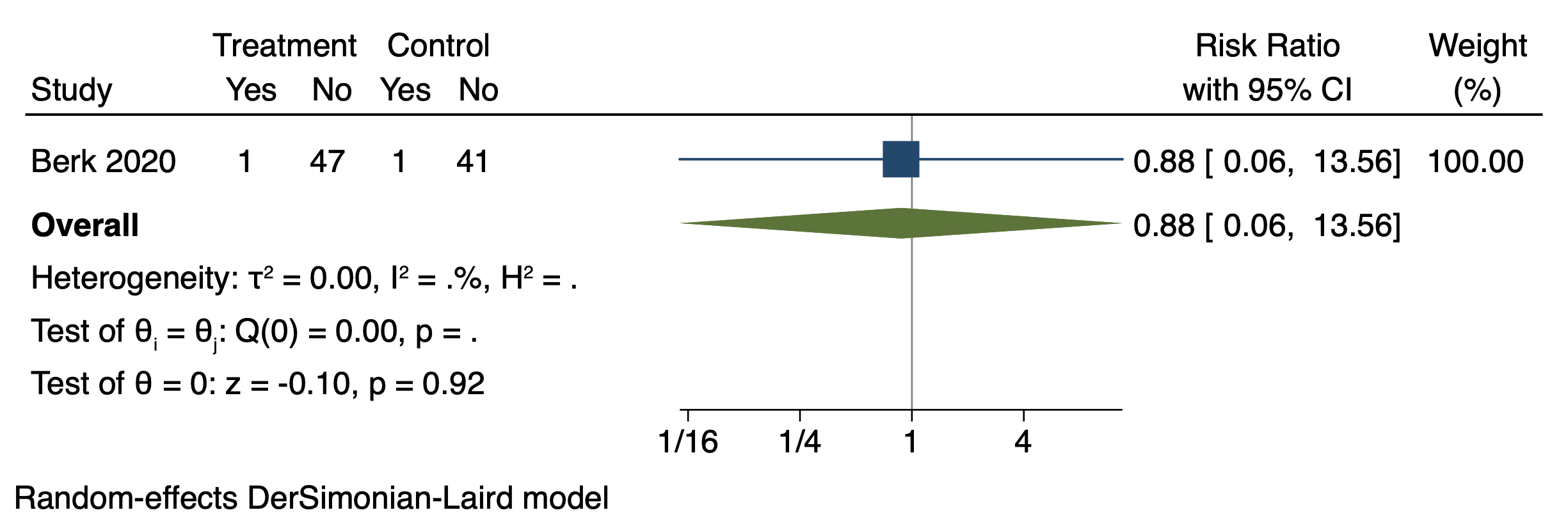
**

*Side-effects, Sweating*

**
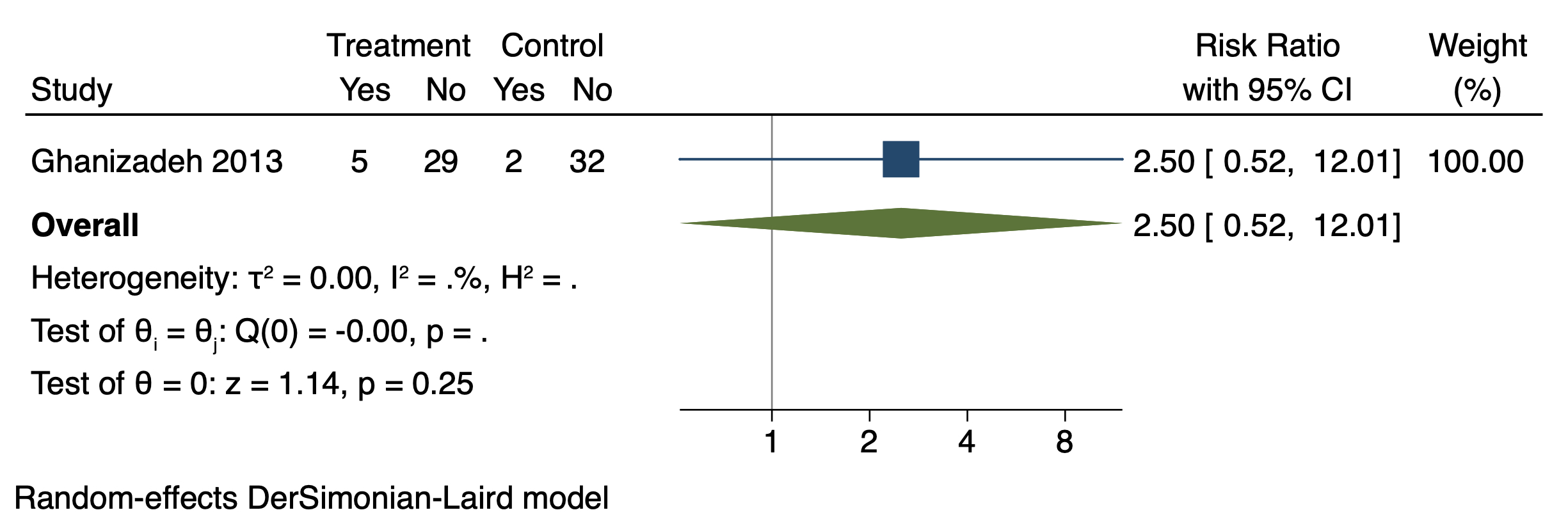
**

*Side-effects, Vomiting*

**
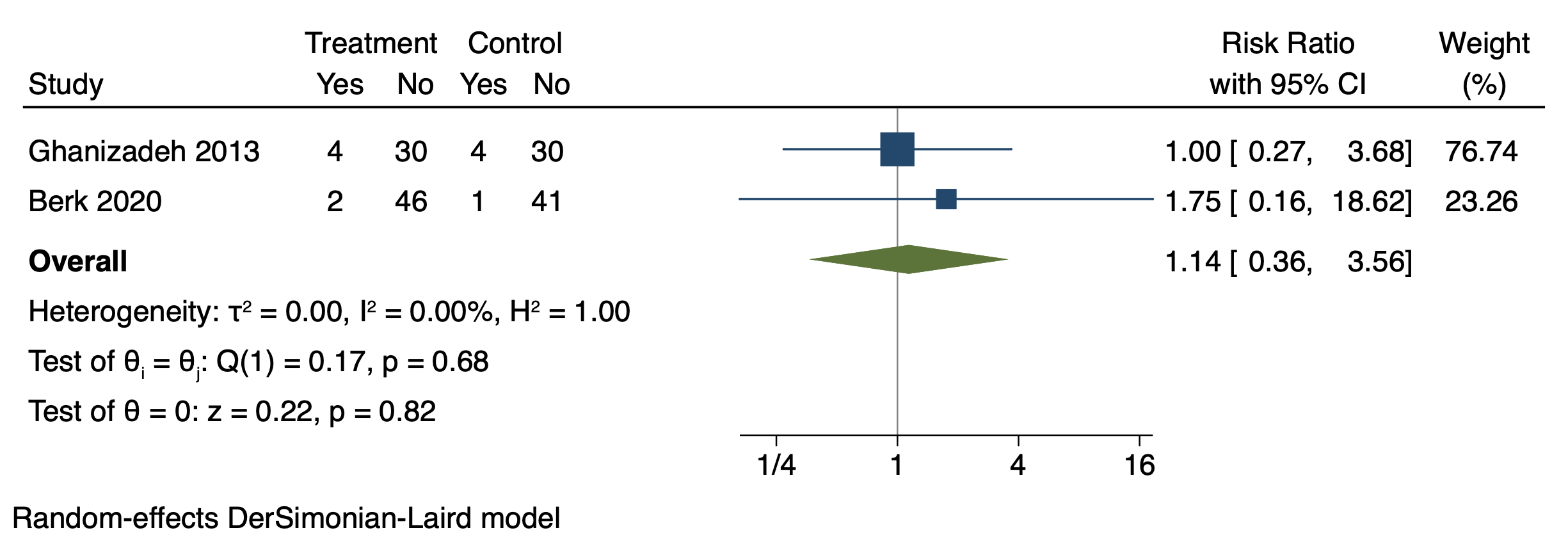
**

**S2 Table - Risk of bias (quality) assessment table**

| ***Risk of Bias*** | **Ghanizadeh 2013**^(1)^ | **Haghighi 2014**^(2)^ | **Gougol 2015**^(3)^ | **Abbasi 2016**^(5)^ | **Berk 2020**^(4)^ |
| --- | --- | --- | --- | --- | --- |
| **Random sequence generation (selection bias)** | The allocation sequence was according to a list provided by a random number generator | The randomization of box numbering was made via a computer random generator | A randomized code was generated by the permuted randomization block method (Excel software). An independent team was involved in the process | Randomization was conducted by using a computerized random number generator (blocks of four, allocation ratio 1:1) | Participants were randomized according to the Inter- national Council for Harmonisation (ICH) Guidelines by an independent researcher |
| **Allocation concealment (selection bias)** | Not reported | The allocation procedure was done with sequentially numbered, identical boxes. The personnel who packaged and prepared the boxes was not further involved in the study | A computer-generated code was used. The personnel who run the randomisation was not involved elsewhere in the project. Assignments were kept in sequentially numbered, sealed, opaque envelopes. They were opened sequentially only after participant details were written on the envelope | An independent party who was not involved elsewhere in the study was responsible for generation of randomisation codes. Concealment of allocation was performed using sequentially numbered, sealed, opaque, and stapled envelopes | Concealed allocation alerts were sent to the local research pharmacists with in- formation regarding participant allocation. The pharma- cist and trial coordinator then ensured that study participants received their assigned study treatment. Re- search coordinators and the clinical team were not aware of allocation. |
| **Blinding of participants and personnel (performance bias)** | Patients and examiners were blind to the allocation groups and lovastatin and placebo tablets were identical; but, as the allocation concealment is not explained, it is not possible to assure that blinding was not broken | Neither patients nor study nurses and physicians were aware of group assignments. Placebo and atorvastatin pills were identical | Sealed envelopes and identical placebo and simvastatin pills were used. The study drugs were dispensed by an investigational drug pharmacist. All members of the research team, patients and their families were blind to the treatment group assignments. They were asked at the end of the study which group they thought they were assigned | The participants, research investigators, rater and the statistician were all blinded to treatment allocation. Simvastatin and atorvastatin tablets were completely identical in their shape, color, size, texture, and odor. Anticipation of the atorvastatin group by the patients and the raters was also evaluated at the study end | All tablets were over-encapsulated for blinding purposes, in order to be identical in appearance and taste.  The study biostatistician and others who were involved in preparing the trial results were blinded to intervention allocation. The trial was only unblinded after finalizing the analysis. |
| **Blinding of outcome assessment (detection bias)** | As the allocation concealment is not explained, it is not possible to assure that blinding was not broken | Personnel were blinded to treatment allocation | Personnel were blinded to treatment allocation | The participants, research investigators, rater and the statistician were all blinded to treatment allocation | The study biostatistician and others who were involved in preparing the trial results were blinded to intervention allocation. The trial was only unblinded after finalizing the analysis. |
| **Incomplete outcome of data (attrition bias)** | Up to 12 % drop-out rate but the missing data was balanced across groups. Reasons were given for drop-outs. Intention-to-treat analysis was used | The attrition rate was 0% | Up to 8.3% drop-out rate but the missing data was balanced across groups. No reason given for drop-outs. The type of analysis used was not described, | 20% drop-out rate balanced across groups. Reasons were given for drop-outs. Intention-to-treat analysis was used |  |
| **Selection reporting (reporting bias)** | The trial was registered at the Iranian Clinical Trials Registry (http://www.irct.ir; IRCT ID: IRCT201304203930N22). However, the trial had been registered only after recruitment had started | The clinical trial registration number is: IRCT201208261743N9; www.irct.ir. | This trial, with registration number IRCT201205131556N43 at the Iranian Registry of Clinical Trial <http://www.irct.ir> | The trial was registered at the Iranian registry of clinical trials prior to conducting the study (<http://www.irct.ir>; registration number: IRCT201410271556N68). However, the trial had been registered only after recruitment had started | The full protocol was registered on the Australian New Zealand Clinical Trials Registry (ACTRN12613000112763) |

##### Green: low risk of bias

Yellow: unclear risk of bias

Red: high risk of bias

**S3 Table- GRADE (certainty) assessment table**

| **Certainty assessment** | | | | | | | **Summary of findings** | | | | |
| --- | --- | --- | --- | --- | --- | --- | --- | --- | --- | --- | --- |
| **Participants  (studies) Follow up** | **Risk of bias** | **Inconsistency** | **Indirectness** | **Imprecision** | **Publication bias** | **Overall certainty of evidence** | **Study event rates (%)** | | **Relative effect (95% CI)** | **Anticipated absolute effects** | |
|  |  |  |  |  |  |  | **With Placebo** | **With Statins** |  | **Risk with Placebo** | **Risk difference with Statins** |
| **Depressive symptoms (follow up: 2 weeks; assessed with: HDRS)** | | | | | | | | | | | |
| 105 (2 RCTs) ^(1,3)^ | not serious | not serious | not serious | very serious ^a^ | none | ⨁⨁◯◯ LOW | 53 | 52 | - | - | SMD **0.18 lower** (0.57 lower to 0.2 higher) |
| **Depressive symptoms (follow up: 4 weeks; assessed with: HDRS)** | | | | | | | | | | | |
| 178 (3 RCTs) ^(2,3,4)^ | not serious | not serious | not serious | very serious ^a^ | none | ⨁⨁◯◯ LOW | 86 | 92 | - | - | SMD **0.22 lower** (0.51 lower to 0.08 higher) |
| **Depressive symptoms (follow up: 8 weeks; assessed with: HDRS)** | | | | | | | | | | | |
| 238 (4 RCTs) ^(1,2,3,4)^ | not serious | not serious | not serious | serious ^b^ | none | ⨁⨁⨁◯ MODERATE | 116 | 122 | - | - | SMD **0.48 lower** (0.74 lower to 0.22 lower) |
| **Depressive symptoms (follow up: 12 weeks; assessed with: HDRS)** | | | | | | | | | | | |
| 134 (2 RCTs) ^(2,4)^ | not serious | serious ^c^ | not serious | serious ^b^ | none | ⨁⨁◯◯ LOW | 64 | 70 | - | - | SMD **0.47 lower** (0.89 lower to 0.05 lower) |
| **Acceptability (follow up: 8 weeks; assessed with: Discontinuation due to any cause)** | | | | | | | | | | | |
| 266 (4 RCTs) ^(1,2,3,4)^ | not serious | not serious | not serious | very serious ^d^ | none | ⨁⨁◯◯ LOW | 13/130 (10.0%) | 14/136 (10.3%) | **RR 0.99** (0.50 to 1.96) | 100 per 1,000 | **1 fewer per 1,000** (from 50 fewer to 96 more) |
| **Tolerability (follow up: 8 weeks; assessed with: Discontinuation due to any adverse event)** | | | | | | | | | | | |
| 266 (4 RCTs) ^(1,2,3,4)^ | not serious | not serious | not serious | very serious ^d^ | none | ⨁⨁◯◯ LOW | 0/130 (0.0%) | 1/136 (0.7%) | **RR 1.40** (0.22 to 8.76) | 0 per 1,000 | **0 fewer per 1,000** (from 0 fewer to 0 fewer) |

**S2 Fig – Network plot for efficacy**


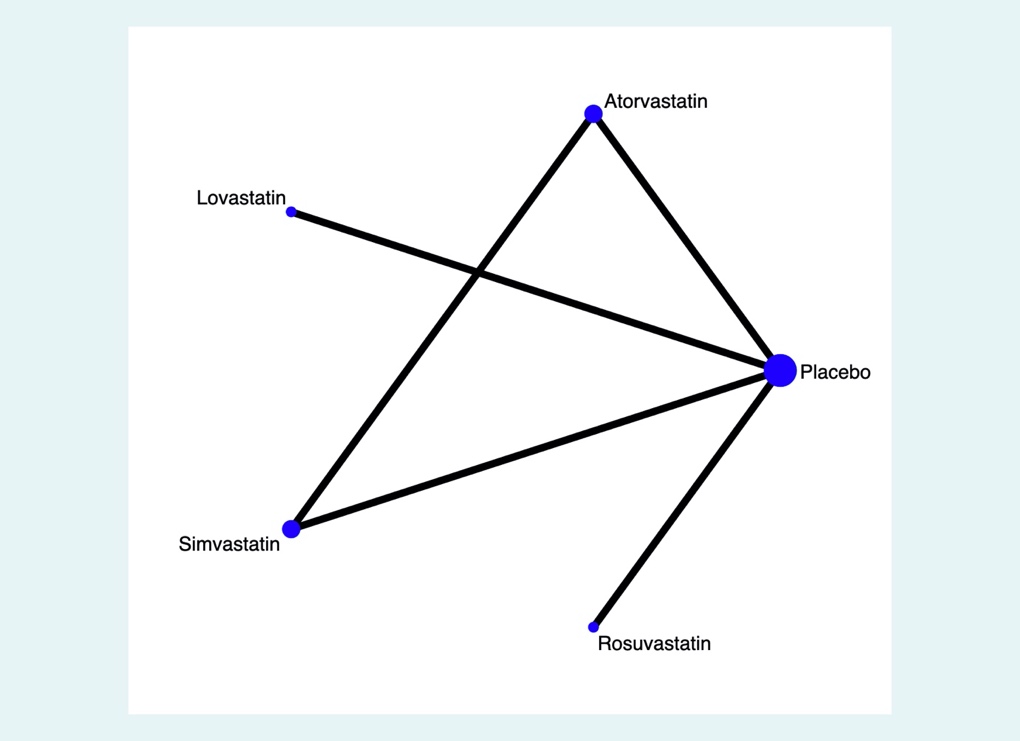


**S4 Table- League table for efficacy (y-axis, green), significative results in bold**

| **Simvastatin** |  |  |  |  |
| --- | --- | --- | --- | --- |
| -0.64 (-1.32 to 0.04) | **Rosuvastatin** |  |  |  |
| **-0.92 (-1.43 to -0.41)** | -0.28 (-0.73 to 0.17) | **Placebo** |  |  |
| -0.16 (-0.91 to 0.59) | 0.49 (-0.23 to 1.20) | **0.77 (0.22 to 1.32)** | **Lovastatin** |  |
| -0.47 (-0.97 to 0.03) | 0.17 (-0.48 to 0.83) | 0.45 (-0.02 to 0.93) | -0.31 (-1.04 to 0.41) | **Atorvastatin** |

**S8 – References**

1. Ghanizadeh A, Hedayati A. Augmentation of fluoxetine with lovastatin for treating major depressive disorder, a randomized double-blind placebo controlled-clinical trial. Depression and Anxiety. 2013;30(11):1084–8.
2. Haghighi M, Holsboer-Trachsler E, Jahangard L, Brand S, Bajoghli H, Ahmadpanah M, et al. In a randomized, double-blind clinical trial, adjuvant atorvastatin improved symptoms of depression and blood lipid values in patients suffering from severe major depressive disorder. Journal of Psychiatric Research [Internet]. 2014;58(2014):109–14. Available from: http://dx.doi.org/10.1016/j.jpsychires.2014.07.018
3. Gougol A, Farokhnia M, Salimi S, Iranpour N, Zareh-Mohammadi N, Yekehtaz H, et al. Simvastatin as an adjuvant therapy to fluoxetine in patients with moderate to severe major depression: A double-blind placebo-controlled trial. Journal of Psychopharmacology. 2015;29(5):575–81.
4. Berk M, Mohebbi M, Dean OM, Cotton SM, Chanen AM, Dodd S, et al. Youth Depression Alleviation with Anti-inflammatory Agents (YoDA-A): a randomized clinical trial of rosuvastatin and aspirin. BMC medicine [Internet]. 2020 Jan 17 [cited 2020 Mar 6];18(1):16. Available from: http://www.ncbi.nlm.nih.gov/pubmed/31948461
5. Abbasi SH, Mohammadinejad P, Shahmansouri N, Salehiomran A, Beglar AA, Zeinoddini A, et al. Simvastatin versus atorvastatin for improving mild to moderate depression in post-coronary artery bypass graft patients: A double-blind, placebo-controlled, randomized trial. Journal of Affective Disorders [Internet]. 2015 Sep 1 [cited 2019 Feb 21];183:149–55. Available from: https://www.sciencedirect.com/science/article/pii/S0165032715002827?via%3Dihub
